# Exploratory Genome-Wide Association Analysis to Identify Pharmacogenetic Determinants of Response to R-CHOP in Diffuse Large B-Cell Lymphoma

**DOI:** 10.3390/cancers15102753

**Published:** 2023-05-13

**Authors:** Gabriele Perrone, Luigi Rigacci, Sara Urru, Sofya Kovalchuk, Marco Brugia, Alberto Fabbri, Lorenzo Iovino, Benedetta Puccini, Emanuele Cencini, Enrico Orciuolo, Silvia Birtolo, Alessandro Melosi, Simone Santini, Ida Landini, Giandomenico Roviello, Raffaella Santi, Alessandra Macciotta, Fulvio Ricceri, Alberto Bosi, Monica Bocchia, Mario Petrini, Enrico Mini, Stefania Nobili

**Affiliations:** 1 Department of Health Sciences, University of Florence, 50139 Florence, Italy; gabriele.perrone@unifi.it (G.P.); ida.landini@unifi.it (I.L.); giandomenico.roviello@unifi.it (G.R.); raffaella.santi@unifi.it (R.S.); 2 DENOTHE Excellence Center, University of Florence, 50139 Florence, Italy; 3 Research Unit of Hematology, Department of Medicine and Surgery, Campus Biomedico University, 00128 Roma, Italy; l.rigacci@policlinicocampus.it; 4 Unit of Biostatistics, Epidemiology and Public Health, Department of Cardiac, Thoracic, Vascular Sciences and Public Health, University of Padova, 35128 Padova, Italy; sara.urru@studenti.unipd.it; 5 Unit of Hematology, Careggi University-Hospital, 50134 Florence, Italy; sofya.kova82@gmail.com (S.K.); puccinib@aou-careggi.toscana.it (B.P.); alberto.bosi@unifi.it (A.B.); 6 Department of Experimental and Clinical Medicine, University of Florence, 50134 Florence, Italy; 7 Unit of Medical Oncology, Careggi University-Hospital, 50134 Florence, Italy; brugiamarco@gmail.com; 8 Unit of Hematology, Azienda Ospedaliera Universitaria Senese, University of Siena, 53100 Siena, Italy; a.fabbri@ao-siena.toscana.it (A.F.); cencioema@libero.it (E.C.); monica.bocchia@unisi.it (M.B.); 9 Unit of Hematology, Santa Chiara University Hospital, University of Pisa, 56100 Pisa, Italy; liovino@fredhutc.org (L.I.); e.orciuolo@ao-pisa.toscana.it (E.O.); mario.petrini@unipi.it (M.P.); 10 Unit of Hematology, Ospedale SS. Cosma e Damiano, 51017 Pescia, Italy; silvia.birtolo@uslcentro.toscana.it; 11 Unit of Hematology, Ospedale San Luca Nuovo, 55100 Lucca, Italy; alemelosi@gmail.com; 12 ASL Toscana Centro, Department of Oncology, Oncohematology Unit, Santo Stefano Hospital, 59100 Prato, Italy; simone.santini@uslcentro.toscana.it; 13 Department of Clinical and Biological Sciences, University of Turin, 10043 Turin, Italy; alessandra.macciotta@unito.it (A.M.); fulvio.ricceri@unito.it (F.R.); 14 Department of Neuroscience, Psychology, Drug Research and Child Health—NEUROFARBA, University of Florence, 50139 Florence, Italy

**Keywords:** diffuse large B-cell lymphoma, R-CHOP, predictive biomarkers, pharmacogenetics, single-nucleotide polymorphism, genome-wide association study

## Abstract

**Simple Summary:**

The standard treatment of patients affected by diffuse large B-cell lymphomas (DLBCLs) is represented by a chemoimmunotherapeutic regimen (R-CHOP) that is successful in about 60% of patients. Currently, we cannot know in advance if their disease will respond to R-CHOP. This study aimed at identifying predictive genetic biomarkers for R-CHOP response through the analysis of a very high number of host genetic polymorphisms. Of the 216 enrolled chemonaïve patients candidate to R-CHOP, 185 were eligible. Highly statistically significant associations were found between progression-free survival and six polymorphisms (i.e., rs116665727, rs1607795, rs75614943, rs77241831, rs117500207, rs78466241), and between the overall survival (OS) and five polymorphisms (i.e., rs74832512, rs117500207, rs35789195, rs11721010, rs12356569). Overall, wild-type patients (i.e., without these polymorphisms) showed prolonged survival compared with polymorphic patients. In the future, these polymorphisms, alone or in combination, after a proper validation in a further number of patients, could contribute to improving the prediction of R-CHOP response.

**Abstract:**

R-CHOP standard chemotherapy is successful in about 60% of diffuse large B-cell lymphoma (DLBCL) patients. Unresponsive patients have a poor prognosis, and predictive biomarkers of response to R-CHOP are lacking. We conducted the first prospective GWAS study aimed at exploring constitutional biomarkers predictive of R-CHOP efficacy and toxicity. Overall, 216 any-stage chemonaïve DLBCL patients candidate to R-CHOP were enrolled. The median age of the 185 eligible patients was 59.2 years, 49.7% were women and 45.4% were stage I–II patients. According to the Revised International Prognostic Index (R-IPI), 14.1%, 56.8% and 29.2% were in the very good, good and poor prognosis groups, respectively. Of the patients, 85.9% produced a complete response. Highly significant associations (i.e., *p* < 5 × 10^−8^) were found between progression-free survival (PFS) and six SNPs (i.e., rs116665727, rs1607795, rs75614943, rs77241831, rs117500207, rs78466241). Additionally, five SNPs (i.e., rs74832512, rs117500207, rs35789195, rs11721010, rs12356569) were highly associated with overall survival (OS). Wild-type patients showed a prolonged PFS or OS compared with patients carrying deleterious alleles (*p* < 0.001). No association with the adequate significant threshold was observed between SNPs and the objective response or toxicity. In the future, these SNPs, alone or in combination, after a proper validation in an independent cohort, could contribute to improving the prediction of R-CHOP response.

## 1. Introduction

Diffuse large B-cell lymphoma (DLBCL) is the most common type of adult non-Hodgkin’s lymphoma, representing about 40% of all newly diagnosed and 90% of aggressive lymphomas [1,2]. DLBCL is a highly heterogeneous disease [3]. Gene expression profiling (GEP) seminal studies identified categories according to the ‘cell-of-origin’ principle, that may help to stratify DLBCL patients with different prognoses [3,4,5,6]. Germinal centre B-cell-like (GCB) DLBCL patients have a significantly better overall survival than those with an activated B-cell-like (ABC) DLBCL. A recent GEP study [7] identified, at the single cell level, GCB cell subpopulations characterized by different signatures, thus providing more detailed assignments of GCB DLBCL able to recognize additional prognostic subgroups. In clinical practice, GEPs are replaced by immunohistochemical algorithms [8,9,10], although their prognostic predictive power is lower compared to GEP [9]. Studies on the genetics of tumour cells have identified further sub-sets of DLBCLs characterized by different alterations and outcomes [11]. To date, the main recognized poor-prognosis genetic subtypes display tumour *MYC* and *BCL2* translocations (‘double-hit lymphoma’) or *MYC*, *BCL2* and *BCL6* translocations (‘triple-hit lymphoma) [12,13,14].

Currently, a DLBCL outcome is clinically predicted by the International Prognostic Index (IPI) that has been revised (R-IPI) after the addition of the anti-CD20 monoclonal antibody rituximab to the CHOP regimen (doxorubicin, cyclophosphamide, vincristine, prednisone) [15].

These prognostic biomarkers are useful to indicate long-term clinical outcomes for DLBCL patients, but they are not informative of the likelihood of patients to respond to R-CHOP in relation to the sensitivity/resistance of the tumour to the drug treatment.

Although R-CHOP significantly improved the outcomes of DLBCL patients compared to CHOP alone [16], about 40% of DLBCL patients, due to tumour heterogeneity, is refractory or relapses after initial complete remission, and most of them ultimately die, with only a minority cured by the salvage therapy [17]. Thus, DLBCL patients would require personalized treatment. However, at present, based on the fundamental clinical trials performed at the beginning of the rituximab era [18,19,20], of which the results were successively confirmed in long-term studies [21,22], the R-CHOP regimen represents the upfront standard of care for all DLBCL stages and for both GCB and non-GCB subtypes [23]. Very recently, the modified regimen R-CHOP, represented by pola-R-CHP in which vincristine is replaced with the anti-CD79b antibody drug conjugate polatuzumab vedotin [24], has been approved for the treatment of previously untreated DLBCL patients. A more aggressive first-line treatment is only recommended for the double/triple-hit lymphomas [25,26]. The results of the “frontMIND” phase III trial that compares an intensified treatment (i.e., the anti-CD19 tafasitamab monoclonal antibody *plus* lenalidomide *plus* R-CHOP vs. R-CHOP) in newly diagnosed high-intermediate and high-risk DLBCL patients, will only be available in the next years [27]. Tafasitamab and other anti-CD19 monoclonal antibodies, as well as the anti-CD19 CAR T-cell therapy, are currently approved only for the treatment of patients with recurrent or refractory DLBCL.

Overall, although a large amount of information is available on the mutational landscape of DLBCL [14,28], recently integrated by an elegant study on genotyping of DLBCL mutations on liquid biopsies [29], less information is available for host genetics in DLBCL.

Germline genetic variations are good candidates to characterize the role of the host in predicting response to drug treatments (antitumor activity, efficacy and host toxicity), since they are constitutional, stable over time and easy for evaluating. Compared with somatic mutations, host genetic variations are not affected by tissue heterogeneity and/or by the evolution of molecular changes that may ultimately lead to changes in molecular profiles within the tumour or during the different stages of tumour development. Pharmacogenetics is, in fact, a recognized discipline for predicting drug response in several tumour types [30], including DLBC [31]. According to a candidate gene-based approach, genes involved in pharmacokinetics/pharmacodynamics (PK/PD) of drugs used in DLBCL or genes associated with the immune function were found to be associated with R-CHOP efficacy [31,32]. However, GWAS represents a hypothesis-free approach that increases the chance of identifying genetic variants in novel genes that cannot be easily included in candidate gene-based approaches. These genes may be involved in several relevant molecular processes comprising drug action and disposition, tumour drug resistance and pathobiology [33], and thus play a potential role in drug response. On this basis, a number of GWAS studies have been performed to identify novel associations between drug efficacy and genetic variants in several cancers, including breast cancer [34], lung cancer [35,36], pancreatic cancer [37], renal cell carcinoma [38], hepatocellular carcinoma [39], as well as DLBCL [40].

To date, only one GWAS analysis has explored the constitutional DNA in DLBCL patients to identify associations between SNPs and clinical outcomes [40], although patients analysed were not uniformly treated.

Thus, due to the substantial lack of data on constitutional biomarkers potentially predictive of response to R-CHOP in DLBCL patients, we designed an exploratory multicentre prospective pharmacogenetic study, based on GWAS analysis. The study primary endpoint was the identification of potential associations between polymorphisms and progression-free survival (PFS) or toxicity. The secondary endpoint was the identification of potential associations between polymorphisms and the overall survival (OS) or objective response.

## 2. Materials and Methods

### 2.1. Participants and Treatment

Study participants were enrolled in this GWAS prospective observational clinical trial from November 2012 to October 2017 at six Divisions of Haematology belonging to Italian university hospitals and first-level hospitals. The inclusion criteria were: signed informed consent; histologically confirmed diagnosis of DLBCL according to WHO classification systems [41,42]; age 18–75 years; PS ECOG ≤ 2; patients eligible for standard R-CHOP; no previous chemotherapy and radiotherapy; availability of blood samples. Exclusion criteria were: a different diagnosis from DLBCL; liver or renal disfunction; previous neoplasms; contraindications for doxorubicin or vincristine treatment. The patient’s prognosis was evaluated according to the Revised IPI (R-IPI) [15] and patients were classified in the following three prognostic groups: R-IPI 0, R-IPI 1–2, R-IPI 3–5, the R-IPI 0 and R-IPI 3–5 groups being those with a more favourable and unfavourable prognosis, respectively. Overall, 216 chemonaïve DLBCL patients (Ann Arbor stages I–IV) eligible for R-CHOP treatment were enrolled. A total of 187 patients were evaluable based on clinical, pathological and genetic information.

R-CHOP was administered as follows: rituximab 375 mg/m^2^ on day 0, cyclophosphamide 750 mg/m^2^ on day 1, doxorubicin 50 mg/m^2^ on day 1, vincristine 1.4 mg/m^2^ (max. 2 mg) on day 1, prednisone 50 mg/m^2^ days 1–5, every 21 days for 6 cycles.

The study was approved by the local IRB (Careggi University Hospital, Florence, Italy) of the coordinator centre (Prot. 2012/0033535) and by those of the participant centres. All patients provided written informed consent.

### 2.2. DNA Extraction and Genotyping Platform

Peripheral whole-blood samples were collected in EDTA vacutainer tubes before the first cycle of R-CHOP. Genomic DNA was extracted from peripheral blood according to standard procedures, by using the Puregene Blood Core Kit (Qiagen, Hilden, Germany). The DNA concentration and quality were evaluated with a Qubit 3.0 Fluorometer. The DNA integrity was assessed with an Agilent 2100 Bioanalyzer.

According to an Affymetrix-based methodology, total gDNA was amplified and, after fragmentation and hybridization, genotyped using the UK Biobank Axiom Array on a GeneTitan MC System INTL instrument (Thermo Fisher, Waltham, MA, USA). The array contained 820,967 SNPs. In this system, GWAS markers were selected using Affymetrix’ imputation-aware marker choice algorithms to provide the GW coverage of common (estimated minor allele frequency (EMAF) ≥ 5%) and low-frequency (1% < EMAF) markers. Array data were uploaded on the NCBI GEO repository as GSE186441.

### 2.3. Bioinformatic Analysis

Bioinformatic analysis to identify host genetic biomarkers associated with R-CHOP efficacy or toxicity was performed. A total of 187 study samples were analysed. Files of .CEL type were imported in the Axiom Analysis Suite (4.0.3 software) (Thermo Fisher) [43] and processed using the Axiom UKB WCSG array type to evaluate sample and SNP quality.

Four quality metrics were used to assess the plate and sample quality [44]: the plate pass rate (PPR) and average plate control rate (APCR) filters were applied to plates, whereas the dish quality control (DQC) and quality control call rate (QCCR) metrics were used to evaluate the sample quality (Supplementary Materials).

Confidence scores (CS) defined as CS = 1 − *p*, where *p* is the posterior probability that the point belongs to the assigned genotype cluster, were calculated to assess the genotype call quality. The CS ranges in [0, 1] and values above the 0.15 threshold generate No Calls.

Sex was automatically assessed by the analysis tool. In the case of unknown sex assignment, X and Y chromosome intensities and heterozygosity rates were checked.

Allele calling and quality control of the genotyping data were performed on a total of 836,727 probesets. In the case of more than one probeset interrogating the same SNP, the best probeset was chosen according to Axiom Analysis Suite metrics, and then the SNP quality was checked. In particular, monomorphic SNPs, SNPs with a call rate < 95%, minor allele frequency < 1%, and those that significantly deviated (*p* < 10^−10^) from the Hardy–Weinberg equilibrium were excluded. To evaluate the SNP quality, Fisher’s linear discriminant, homozygous ratio offset and heterozygous strength offset default thresholds were also considered. Principal components analysis was performed to identify the eventual substructure in the population. Finally preliminary association tests were carried out considering only the samples and SNPs passing quality filters.

### 2.4. Patient Population and Quality Control of Genotypic Data

Of the 216 patients enrolled, 13 were not evaluable due to the lack of sufficient clinical information. In addition, 3 patients who interrupted the treatment early, 2 patients who underwent a treatment other than the R-CHOP regimen, 1 who was affected by follicular lymphoma, and 2 patients who were not of Caucasian origin, were excluded. Eight patients were excluded due to sample and/or DNA degradation. A total of 187 study samples were analysed, considering ~800,000 probesets.

Study samples were distributed into four plates: three were defined as being of high quality because of PPR ≥ 95% and APCR > 99%, the remaining one had PPR = 92.31%. The DQC and QCCR metrics were used to evaluate the sample quality and, because of the low QCCR (<97%), two samples were excluded from the subsequent analysis. The median CS was in the order of 10^−5^, confirming the high-quality genotype calls.

Two samples were identified with an unknown sex, so X and Y chromosome intensities and heterozygosity rates were checked. This analysis led to the male sex for both samples, in agreement with the collected data. PCA identified two components which were used to adjust the association tests.

### 2.5. R-CHOP Efficacy and Toxicity Evaluation

Objective response was evaluated during (e.g., third cycle) and at the end (sixth cycle) of the R-CHOP treatment by the Cheson standardized response criteria [45] until 2014, and subsequently by the Cheson revised response criteria [46]. According to the Cheson criteria, the treatment response was classified as complete remission (CR), partial remission (PR), stable disease (SD) and progression disease (PD). The PFS duration was calculated from the date of diagnosis until the PD or last follow-up, whereas the OS duration from the date of the diagnosis until death or the last follow-up.

The R-CHOP toxicity was evaluated according to the NCI-CTCAE version 4.03 criteria.

### 2.6. Statistical Analysis

Treatment efficacy was measured *via* PFS, OS and objective response.

To identify correlations between clinical/pathological characteristics or SNPs and the study endpoints, Cox (PFS and OS) and logistic regressions (objective response) were used. For correlation studies, patients were grouped as complete response (CR) and not CR (i.e., partial response, stable disease, progression) patients. The age was analysed as a continuous variable.

Five types of toxicity were considered as a coprimary endpoint: neutropenia, haematological toxicity, gastrointestinal toxicity, infection and maximum toxicity (i.e., the highest G among the five study types of toxicity). To test the SNP association, two logistic regression models were performed for every type of toxicity: the first one considered toxicity (grades from 1 to 4, i.e., G1–G4) versus no toxicity (grade 0, i.e., G0), and the second considered severe toxicity (grades 3 and 4, i.e., G3–G4) versus low toxicity (grades 0, 1 and 2, i.e., G0–G2).

The models were then adjusted for principal components, sex, R-IPI and all of them and Manhattan plots were generated to show the association results. The models adjusted for age and stage were not considered, as these parameters were included in the R-IPI.

GWAS analysis were performed using the GWASTools package of the statistical software R version 4.0.2 (R Core Team (2020)—https://www.r-project.org/, accessed on 15 December 2022).

The Kaplan–Meier survival plots were generated for the SNPs that were found to be associated with PFS and/or OS through bioinformatic analysis with *p*-values < 5 × 10^−8^. *p* < 0.05 was considered statistically significant; the statistical analysis was performed using the SPSS software v.26 (IBM, Armonk, NY, USA).

### 2.7. Functional Enrichment and Network Analysis

The lists of SNPs associated with the PFS or OS, with or without adjustment for sex and/or R-IPI, were uploaded into the DAVID bioinformatics tool (https://david.ncifcrf.gov/, accessed on16 January 2023) to perform functional enrichment analysis, or into Metacore^TM^ to perform network analysis. The latter is a web-based bioinformatics suite that allows researchers to upload data analysis results from genomic experiments and provides functional analysis to identify the most relevant pathways, networks and cellular processes in the data. The shortest path function was applied allowing for one intermediary to connect the uploaded elements.

## 3. Results

### 3.1. Relationships between Efficacy Parameters and Clinical/Pathological Characteristics

Overall, 185 patients were evaluable. Clinical and pathological characteristics are listed in Table 1. Sex was well balanced (i.e., 50.3% men). At presentation, 45.4% of patients were early-stage (stage I–II), 70.8% had good or very good R-IPI. The performance status was 0 in 62.2% of the cases. The median follow-up was 45 months, and the median PFS and OS were not reached at the time of the analysis. The objective response was observed in 176 patients (95.1%) (i.e., CR 89%, *n* = 159; PR 9.2%, *n* = 17). One patient (0.5%) showed SD and eight patients (4.3%) showed PD.

**Figure 1:**
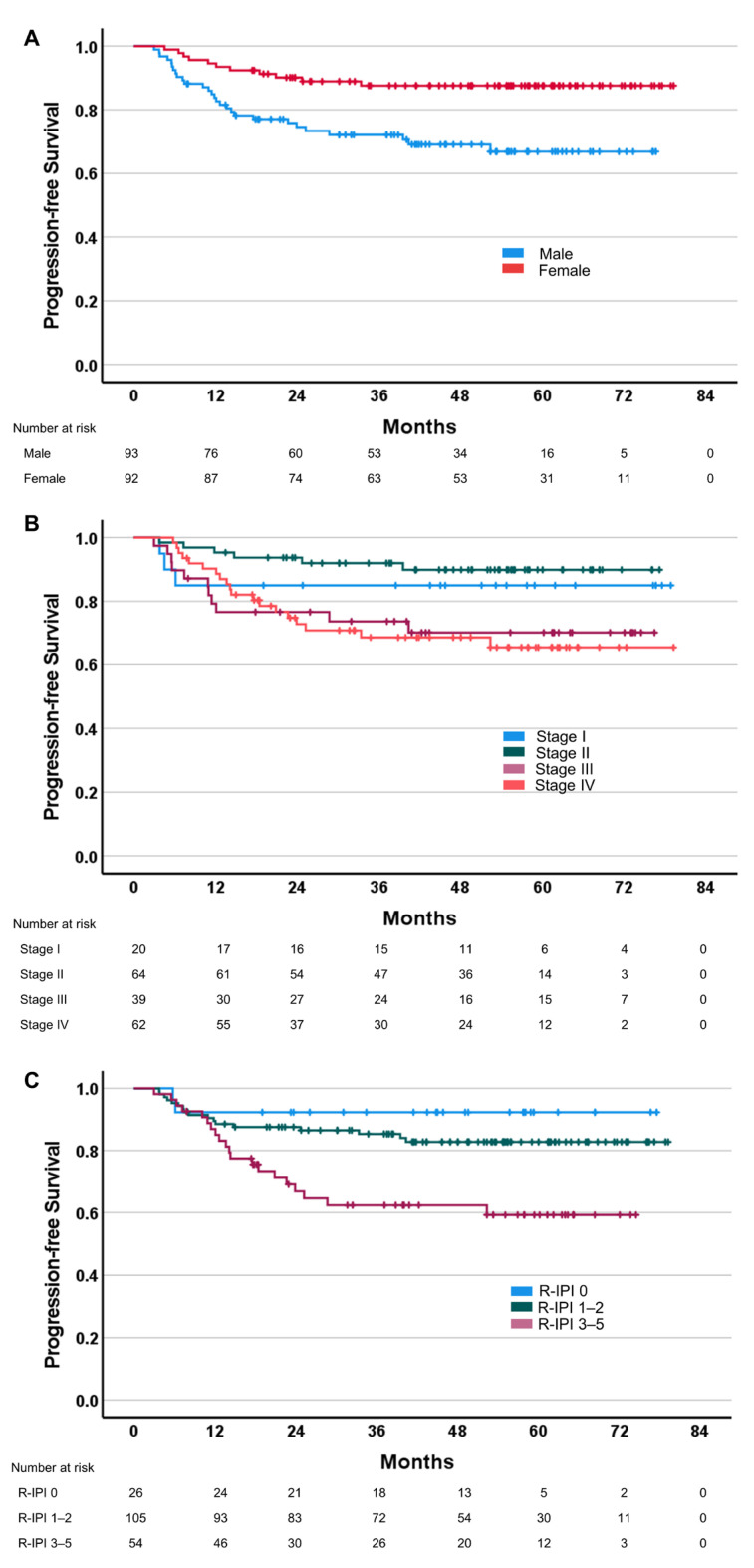
Kaplan–Meyer curves of progression-free survival according to the clinical/pathological characteristics of DLBCL patients (*n* = 185). (**A**) Sex (log rank test, *p* = 0.002; Cox regression: HR 0.34; CI 0.17–0.69; *p* = 0.003); (**B**) stage (log rank test, *p* = 0.016; Cox regression: HR 3.12; CI 1.48–6.59; *p* = 0.003); (**C**) R-IPI (log rank test, *p* = 0.002; Cox regression: HR 2.55; CI 1.34–4.88; *p* = 0.005).

According to Cox regression analysis, PFS was significantly associated with sex (more prolonged for female), R-IPI (shorter for R-IPI 3–5) and stage (shorter for stage III–IV) (Figure 1). The OS was associated with sex (i.e., prolonged in females), age (i.e., shorter in older patients), R-IPI (shorter for R-IPI 3–5) and stage (shorter for stage III–IV). No other clinical parameter was found to be associated with the PFS or OS. The objective response was significantly associated with the stage and R-IPI; in particular, stage III–IV and R-IPI-poor patients had a worse response (odds ratio (OR) 0.39, CI 0.15–0.95, *p* = 0.046 and OR 0.37, CI 0.14–0.92, *p* = 0.032, respectively). No significant association was found between the objective response and sex or age (Supplementary Materials).

### 3.2. Relationships between Efficacy Parameters and SNPs

Overall, highly significant associations (i.e., *p* < 5 × 10^–8^) were found between PFS and six SNPs (i.e., rs116665727, rs1607795, rs75614943, rs77241831, rs117500207 and rs78466241) after R-IPI and/or sex-unadjusted or -adjusted analyses. Similarly, five SNPs (i.e., rs74832512, rs117500207, rs35789195, rs11721010 and rs12356569) were highly associated with the OS after the same unadjusted or adjusted analyses (Supplementary Materials). Wild-type patients showed a prolonged PFS and OS compared with patients carrying deleterious alleles (*p* < 0.001). No association with the adequate significant threshold was observed between the SNPs and the objective response, with the *p*-value of the highest associations in the order of 10^−5^ (Supplementary Materials).

#### 3.2.1. PFS Associations

**Figure 2:**
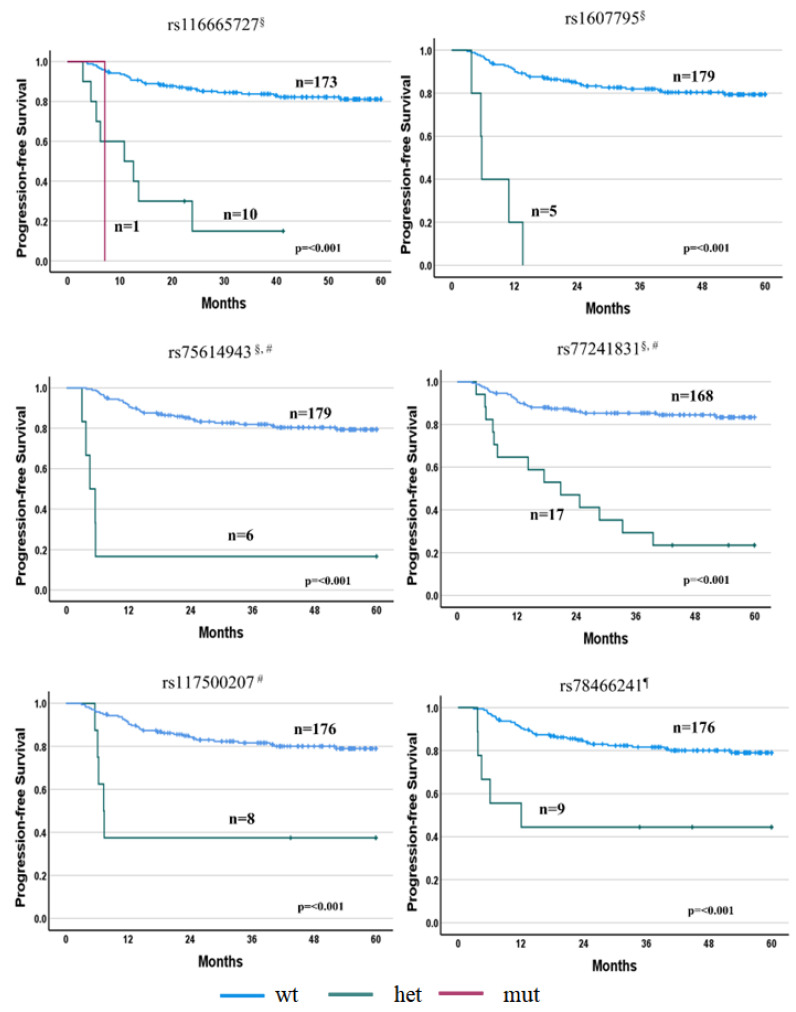
Progression-free survival according to the SNPs that reached the adequate GWAS statistical significance (*p* < 5 × 10^–8^). Wild-type patients showed a prolonged PFS compared with polymorphic patients. ^§^ Unadjusted analysis; ^#^ adjusted for R-IPI; ^¶^ adjusted for sex and R-IPI.

Preliminary association tests (i.e., unadjusted analysis) with the PFS identified four SNPs reaching the significance threshold (*p* < 5 × 10^–8^): rs116665727 in the *TNIP3* gene (HR = 6.77, *p* = 1.0 × 10^−9^), rs1607795 in the *LNC00882* gene (HR = 18.56, *p* = 1.3 × 10^−8^), rs75614943 in the *CIDEA* gene (HR = 15.73, *p* = 1.9 × 10^−8^) and rs77241831 in the *NOS1* gene (HR = 6.66, *p* = 3.1 × 10^−8^). Twelve further SNPs were identified with a lower, but still relevant, significant association level (from *p* ≥ 5 × 10^−8^ to *p* = 10^−7^) (Figure 2; Supplementary Materials).

Two out of these four SNPs, i.e., rs75614943 in the *CIDEA* gene (HR = 20.67, *p* = 7.0 × 10^−9^) and rs77241831 in the *NOS1* gene (HR = 6.58, *p* = 4.5 × 10^−8^) were also highly associated with the PFS after R-IPI adjustment. In addition to SNPs in the *CIDEA* and *NOS1* genes, a third SNP, i.e., rs117500207 in the *PPP6R3* gene, was found to be associated with the PFS after R-IPI adjustment (HR = 24.38, *p* = 2.6 × 10^−8^) (Figure 2; Supplementary Materials).

Two additional SNPs, rs1607795 in the *LNC00882* and rs74832512 in the *SLC35F4* gene, were exactly on (HR = 15.98, *p* = 5.0 × 10^−8^) and just above (HR = 30.69, *p* = 7.9 × 10^−8^) the threshold, respectively (Figure 2 and Supplementary Materials), after adjustment for R-IPI. A further nine SNPs were associated with *p*-values ranging from 5x10^−8^ to 10^−7^.

Adjusting the analysis for R-IPI and sex, one SNP was highly statistically associated with the PFS, i.e., the rs78466241 in *CSRP3-AS1* intron variant (HR = 32.42, *p* = 6.0 × 10^−9^) (Figure 2 and Supplementary Materials), whereas two further SNPs, i.e., rs12356569 in the *LYZL1* (HR = 27.64, *p* = 5.1 × 10^−8^) and rs68362253 in the *PPP6R3* (HR = 22.08, *p* = 6.8 × 10^−8^) genes, were just above the threshold (Supplementary Materials).

#### 3.2.2. OS Associations

**Figure 3:**
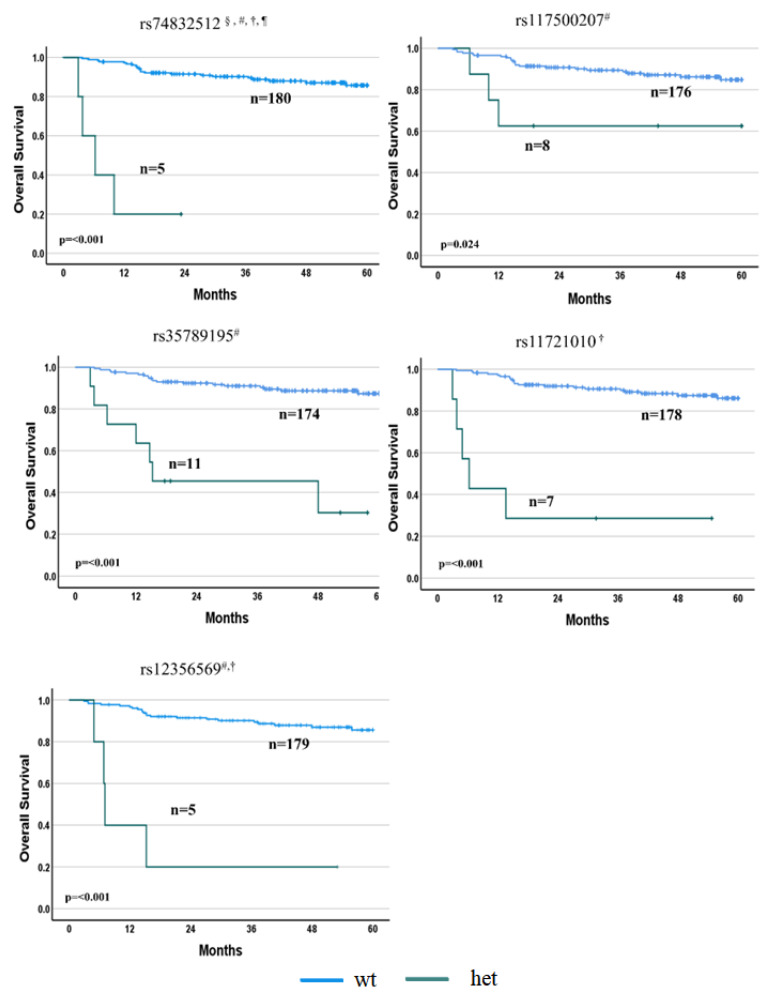
Overall survival according to the SNPs that reached the adequate GWAS statistical significance (*p* < 5 × 10^–8^). Wild-type patients showed a prolonged OS compared with polymorphic patients. ^§^ Unadjusted analysis; ^#^ adjusted for R-IPI; ^†^ adjusted for sex; ^¶^ adjusted for sex and R-IPI.

By preliminary association tests between the OS and SNPs, only one SNP reached the significance threshold: rs74832512 in the *SLC35F4* gene (HR = 26.48, *p* = 1.7 × 10^−8^). This result was also confirmed by adjusting the analysis for R-IPI (HR = 56.03, *p* = 7.0 × 10^−9^), sex (HR = 30.63, *p* = 2.3 × 10^−8^) and R-IPI *plus* sex (HR = 53.91, *p* = 3.4 × 10^–8^) (Figure 3 and Supplementary Materials). After adjustment for sex, the association between rs11721010 in the *OXNAD1* gene and the OS was also highly significant (HR = 20.30; *p* = 2.7 × 10^−8^) (Figure 3 and Supplementary Materials).

Among the SNPs associated with the OS after adjusting for R-IPI, in addition to rs74832512 in the *SLC35F4* gene, two other SNPs showed an adequate GWAS *p*-value: rs117500207 in the *PPP6R3* (HR = 25.46, *p* = 2.6 × 10^−8^) and rs35789195 in the *CDH3* genes (HR = 9.65, *p* = 4.9 × 10^−8^). Just below the threshold, a further SNP, rs61759901 on gene *RGS11* (HR = 57.30, *p* = 5.1 × 10^−8^), was associated with the OS (Figure 3 and Supplementary Materials).

When the analysis was adjusted for R-IPI and sex, in addition to rs74832512 in the *SLC35F4* gene, a further SNP, i.e., rs12356569 in the *LYZL1* gene (HR = 39.70, *p* = 4.0 × 10^−8^), was identified with adequate statistical significance (Figure 3 and Supplementary Materials). Interestingly, among the five SNPs significantly associated with the OS, in addition to rs117500207 in the *PPP6R3* gene that was also significantly associated (*p* < 5 × 10^−8^) with the PFS, rs74832512 in the *SLC35F4* and rs12356569 in the *LYZL1* genes were associated with the PFS, with a *p*-value > 5 × 10^−8^–10^−7^. rs35789195 in the *CDH3* and rs11721010 in the *OXNAD1* genes were associated with the PFS, with *p*-values of 1.3 × 10^−6^ and 1.4 × 10^−6^, respectively (Supplementary Materials).

### 3.3. Relationships between Toxicity and Clinical/Pathological Characteristics

Overall, G1–4 toxicity was reported in 80.5% of the patients. The comparisons between G0–2 and G3–4 total toxicity, neutropenia, hematological toxicity showed an association with age with a higher risk in older patients compared with younger patients (*p* < 0.01). The comparison between G0 and G1–4 toxicity confirmed the association with age (*p* = 0.004) (Supplementary Materials).

### 3.4. Relationships between Toxicity and SNPs

When preliminary association tests were performed comparing G0 against G1–4 toxicity, the smaller *p*-value was 10^−5^. The same level of association was observed when adjusting for sex or R-IPI, whereas adjusting for R-IPI and sex, the smaller *p*-value was 10^−6^ (Supplementary Materials).

When the association analysis was performed between toxicity (G0–2 vs. G3–4) and SNPs, no SNP reached the requested GWAS statistical significance, with the smaller *p*-values around 10^−6^, even after sex and/or R-IPI corrections (Supplementary Materials).

No association was observed between the SNPs and hematological toxicity, gastrointestinal toxicity or infection.

### 3.5. Functional Enrichment and Network Analysis

**Figure 4:**
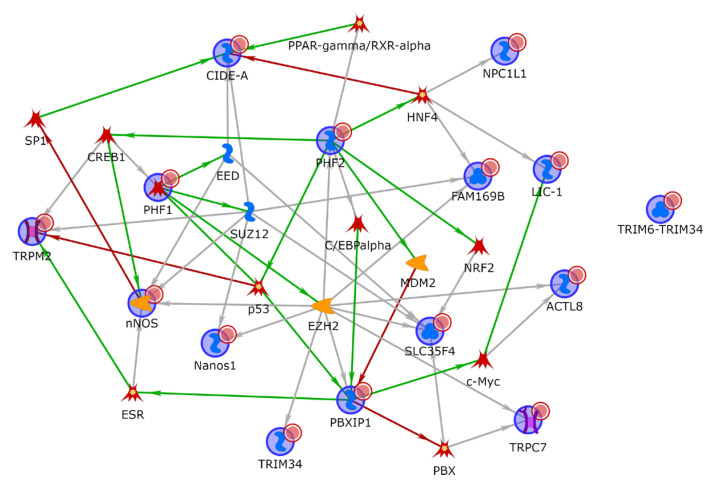
Network analysis of genes of which the SNPs were associated to the PFS, with *p*-values lower than 10^−7^ (genes included in the analysis: *TNIP3*, *CIDEA*, *NOS1*, *NPC1L1*, *DYNC1LI1*, *PBXIP1*, *PHF2*, *LOC339568*, *SLC35F4*, *TRIM34*, *TRPC7*, *ACTL8*, *FAM169B*, *LOC101927873*) (MetacoreTM software).

To better characterize the biological role of the genes linked to the SNPs associated with survival, functional enrichment and network analysis were performed taking into consideration not only genes of which the associations reached *p* < 5 × 10^−8^, but also those reaching *p* < 10^−7^. Overall, 14 and 13 genes were considered for PFS and OS, respectively. The list of genes linked to PFS-associated SNPs is mostly enriched in the regulation of chromosome organization (GO:0033044, with *PHF2*, *DYNC1LI*, *NOS1*), histone modifications (GO:0016570, with *PHF2*, *PBXIP1* and *NOS1*), the regulation of nucleobase-containing compound metabolic process (GO:0019219, with *PHF2*, *TNIP3*, *CIDEA*, *PBXIP1*, *NOS1*, *TRIM34)* and processes related with the positive regulation of transcription, with the *PHF2*, *TNIP3*, *PBXIP1* and *NOS1* genes. Interestingly, the phagosome pathway was overrepresented as well, with the *DYNC1LI1* and *NOS1* genes. Gene network analysis also revealed that all 14 genes, except for *TRIM34*, are interconnected by addition of only one intermediary, with *PHF2* and *PBXIP1* being the most interconnected (Figure 4).

Only one GO biological process was overrepresented within the list of genes linked to OS-associated SNPs: the regulation of signal transduction (GO:0009966, with *CDH3*, *AFAP1*, *RGS11*, *NOS1*, *TRIM34*).

## 4. Discussion

The high heterogeneity of DLBCL that has been shown at genetic, transcriptomic and immunophenotypic levels, reflects the variability in DLBCL prognosis and drug response [14]. However, despite this knowledge, the prediction of prognosis for DLBCL is mainly based on IPI that includes clinical, pathological and haemato-chemical factors, but not molecular factors. Thus, integrating the current factors with molecular factors could improve the predictive value of IPI. To date, the analysis of specific biomarkers of prognosis drives the treatment of double/triple-hit lymphoma patients, to whom more aggressive treatments (e.g., dose-adjusted EPOCH-R—etoposide, prednisone, vincristine, cyclophosphamide, doxorubicin, and rituximab) may be offered [23,25]. Otherwise, independently of the IPI score, the R-CHOP regimen is the recommended first-line chemo-immunotherapy for most DLBCL patients, based on its higher efficacy compared with CHOP alone [18,19,20].

Nevertheless, about 40% of patients will relapse after R-CHOP first-line treatment.

To date, molecular prognostic and/or predictive biomarkers have been mainly studied at the tumour level [47,48,49]. Host genetics has been, instead, poorly investigated in terms of DLBCL prognosis and therapeutic outcome. The available studies are substantially based on a candidate gene approach, and few polymorphisms in genes involved in the ADME of R-CHOP drugs or in immunity, inflammation and apoptotic processes have been identified as potential predictors of DLBCL outcomes [31,32]. However, these data, commonly obtained in retrospective studies, need validation in prospective clinical trials. Currently, no prospective trials specifically designed to investigate SNP prediction of R-CHOP outcomes is ongoing (www.clinicaltrials.gov, accessed on 4 May 2023). Today, only one GWAS analysis investigating the relationships between host genetics and immunochemotherapy in DLBCL patients not uniformly treated, is available [40]. It is based on a meta-analysis of GWAS study datasets, in which some associations were identified. The top loci for event-free survival (EFS) were marked by rs7712513 at 5q23.2 (*p* = 2.08 × 10^−7^), and rs7765004 at 6q21 (*p* = 7.09 × 10^−7^). Both SNPs were also associated with OS (*p* = 3.53 × 10^−8^ and 5.36 × 10^−8^, respectively). These authors also performed an exploratory analysis by building a two-SNP risk score that was found to be highly predictive of EFS (*p* = 1.78 × 10^−12^).

Thus, to contribute to the current knowledge on the pharmacogenetics of DLBCL, we designed this exploratory pharmacogenetic prospective multicentre study based on an unsupervised analysis of constitutional SNPs, and aimed at identifying SNPs potentially predictive for R-CHOP efficacy.

Through bioinformatic analysis, we identified a total of six SNPs highly associated (*p* < 5 × 10^−8^) with the PFS (i.e., *TNIP3*, *LNC00882*, *CIDEA*, *NOS1, PPP6R3*, *CSRP3-AS1*) and five SNPs with the OS (i.e., *SLC35F4*, *PPP6R3*, *CDH3*, *OXNAD1*, *LYZL1*). One of them, rs117500207 in the *PPP6R3* gene, was found to be highly associated with both the PFS and OS. Some SNPs highly associated with the PFS were also associated with the OS, although with a slightly higher *p*-value (*p* ≤ 10^−7^) (e.g., *NOS1*), and all the SNPs highly associated with the OS were associated with the PFS with a high trend (i.e., *LYZL1*, *p* = 5 × 10^−8^; *SLC35F4 p* = 7.9 × 10^−8^) or *p*-values equal to 10^−6^ (Supplementary Materials). However, the reason for which only one SNP (i.e., rs117500207 in *PPP6R3*) reached the *p*-value planned cut-off in both PFS and OS may be due to the fact that among the relapsed patients, only 64% had died at the time of the analysis. Thus, the lower number of events observed in OS (death) compared with PFS (progression), may have provided the differences in *p*-values observed for the associations between the same SNPs and the two survival parameters. Among genes of which the SNPs were associated with the clinical outcomes of this cohort of patients, some have been previously related to cancer, including lymphomas (e.g., *CDH3*, *OXNAD1*). However, none of the ten genes of which the SNPs were associated with survival parameters have never been found to be mutated in ctDNA from DLBCL) [50,51,52]. The rs77241831 SNP in the *NOS1* gene was found to be highly associated with the PFS (*p* < 5 × 10^−8^) after unadjusted analysis and R-IPI adjustment. NOS1 belongs to the family of nitric oxide synthases that synthesize nitric oxide (NO) starting from L-arginine. The evaluation of NOS isoform expression levels in B-cell non-Hodgkin lymphoma tissues showed NOS1 more frequently expressed compared with NOS2 and NOS3 (94% of cases vs. 87% vs. 6%, respectively) [53]. Interestingly, some studies highlighted associations between NOS genetic polymorphisms and non-Hodgkin lymphoma risk [54,55].

The SNP, rs75614943, in the cell death inducing DFFA (DNA fragmentation factor subunit Alpha), such as effector A (*CIDEA),* gene was highly associated with PFS after preliminary analysis and R-IPI adjustment (*p* < 5 × 10^−8^). The CIDEA protein has been shown to play a role in lipolysis in animal models and humans [56], and has also been suggested to play a role in human cancer cachexia [57].

As far as the SNPrs116665727 in the *TNIP3* gene is concerned, it was found to be highly associated with the PFS after preliminary analysis (*p* < 5 × 10^−8^). A number of autoimmune diseases have been associated with the TNIP3 protein and related mutations [58].

The remaining three genes of which the SNPs were significantly associated with the PFS were long intergenic non-protein coding RNA 882 (*LNC00882*), cysteine and glycine rich protein 3, and E2F8 antisense RNA 1 (*CSRP3-AS1*) and *PPP6R3*. No information on the biological and/or pathological role of *CSRP3-AS1* is available, while a relationship between *LNC00882* and pathological processes, including cancer (e.g., colorectal cancer) [59] has been suggested.

The rs117500207 SNP in *PPP6R3* gene was found to be highly associated to both the PFS and OS after R-IPI adjustment. PPP6R3 is a regulatory subunit for the PPP6 catalytic subunit that is involved in the turnover of serine and threonine phosphorylation events during mitosis. This protein is ubiquitary-expressed in human tissues and highly expressed in immune cells and lymphoid tissues. The *PPP6R3* gene was recently included in a 95-gene blood transcriptome signature that represents a link between *C. burnetii*, the agent that causes Q fever, and non-Hodgkin lymphoma [60].

In addition to *PPP6R3*, other four genes were significantly associated with the OS, with or without adjustment for R-IPI and/or sex (i.e., rs74832512 in *SLC35F4)*, after adjustment for sex (i.e., rs11721010 in *OXNAD1*), after adjustment for R-IPI (i.e., rs35789195 in *CDH3*), after adjustment for R-IPI and sex (i.e., rs12356560 in *LYZL1*).

The *SLC35F4* gene codifies the SLC35F4 transporter belonging to the solute carrier 35 (SLC35) transporter family. Overall, *SLC35F4* has been poorly investigated. Available information derived from a complex miRNA–mRNA analysis in different lymphoma types, including DLBCL, reports that this gene is targeted by the hsa-miR-4524a-3p through a miRNA–mRNA interaction with negative correlation [61], thus suggesting that this gene is part of the regulatory pathways relevant in lymphomagenesis.

*OXNAD1* (oxidoreductase NAD-binding domain containing 1) gene codifies the oxidoreductase NAD-binding domain-containing protein 1 protein, an ubiquitous protein of which the main expression was detected in lymph nodes. Interestingly, aberrations in this gene (2p24.3) have been found in translocation-positive follicular lymphoma [62]. However, no information is available in relation to the potential role of the *OXNAD1* gene in drug efficacy of lymphomas or other cancers.

The SNP, rs12356569 in the *LYZL1* (lysozyme like 1) gene was found to be highly associated with the OS, and with a very high trend with the PFS (i.e., *p* = 5 × 10^−8^) after adjustment for sex and R-IPI. The expression of the LYZL1 protein has been detected in a small number of cancer tissues, mainly ovarian and liver cancers, and not in lymphomas. Overall, its role in disease or cancer has not been characterized, while the potential role of *LYZL1* in anticancer drug resistance has been suggested. In fact, in a recent study, *LYZL1* gene expression was found to vary according to the expression of two biomarkers that have been suggested to be involved in ovarian cancer paclitaxel resistance (i.e., low *MAD2*/high *TLR4* expression: increased expression of *LYZL1*; high *MAD2*/low *TLR4* expression: decreased expression of *LYZL1*) [63].

The *CDH3* (cadherin 3) gene codifies placenta (P-) cadherin, a Ca^2+^-dependent cell–cell adhesion protein, belonging to the cadherin superfamily. As known, cadherins in addition to their physiological roles [64] also play a recognized role in carcinogenesis, tumour invasion and metastasis [64,65].

In relation to survival parameters, we also performed a network analysis that included all genes associated with survival, with *p* < 10^−7^ (i.e., 14 genes with the PFS and 13 genes with the OS). The aim was to obtain information on the biological processes in which these genes were involved regardless of a forced selection, based on the GWAS *p*-value cut-off (*p* < 5 × 10^−8^) that could underestimate the relevant biological information. This knowledge that informs of the common or related biological functions of genes belonging to the same connected component of the network also allows to highlight the co-regulated genes that could play a role in the clinical outcome also by their potential involvement in the mechanisms of action/resistance of the administered drugs. The biological processes that we identified were mainly related to histone and chromatin modifications, the positive regulation of RNA (i.e., transcription, synthetic and metabolic processes) and the regulation of signal transduction. These observations suggest a crosstalk between signal transduction and chromatin and/or epigenetic modifications that may ultimately reflect changes in RNA levels. Overall, the genes identified could contribute to refine genetically altered transcriptional regulators, as well as epigenetic regulators that, although recognized as relevant in B-cell lymphomas [66], have been substantially poorly investigated.

Finally, no association endowed by the adequate statistical significance was found between the SNPs and toxicity or objective response. However, although the observed side effects were substantially expected, SNPs that have been associated with toxicity with 10^−6^ *p*-values should be further investigated in an independent cohort of patients to better understand their potential role in R-CHOP toxicity independently of the achievement of the GWAS significant *p*-value. The lack of associations with the objective response could be related to the very early determination of this efficacy parameter. In fact, the analysis took into consideration the objective response established at the end of the immunochemotherapy treatment, when DLBCL is usually well controlled.

To the best of our knowledge, this is the first prospective study designed with the aim of evaluating the associations between SNPs and R-CHOP response by GWAS. Thus, it overcomes the potential limits of retrospective analyses. However, we acknowledge some limitations in this study; for instance, the absence of an independent replication data set. This was due to the difficulty to find a cohort of DLBCL patients whose DNA was available, uniformly treated with R-CHOP, with adequate clinical information (treatment efficacy/toxicity data) and a long follow-up. In addition, since only another GWAS study performed in non-Hodgkin lymphoma patients took into consideration pharmacogenetic relationships [40], no data are currently available in public repositories and this also hampered the possibility to carry out an in silico validation. For this reason, a future development of this work could be the enrolment of a new cohort of DLBCL patients to validate the identified SNPs.

## 5. Conclusions

Overall, our exploratory study acquires relevance in the framework of the pharmacological treatment of this tumour that, as mentioned, is currently substantially limited to R-CHOP in the first-line treatment. In our opinion, among the ten identified constitutional genetic variations, those that will be successfully validated in an independent cohort of DLBCL patients treated with R-CHOP could integrate the currently known clinical and tumour genetic/genomic risk factors that are used at present following first-line chemo-immunotherapy, to identify patients candidate to intensified treatments in order to circumvent tumour drug resistance upfront. These SNPs could also be helpful in characterizing the new mechanisms of DLBCL drug resistance and contribute to the implementation of personalized treatments through the use of potential not cross-resistant drug combination regimens.

In conclusion, our exploratory analysis discovered novel SNPs potentially predictive for R-CHOP efficacy in DLBCL patients, thus providing support to the hypothesis that the implementation of pharmacogenetics in DLBCL may contribute to a personalized patient treatment selection in the future.

## Figures and Tables

**Table 1:** Clinical and pathological characteristics of patients.

		No.
No.		185
Age (mean (SD))		59.17 (13.56)
Sex	Male	93 (50.3)
	Female	92 (49.7)
Disease stage (%)	I	20 (10.8)
	II	64 (34.6)
	III	39 (21.1)
	IV	62 (33.5)
R-IPI (%)	Very good (0)	26 (14.1)
	Good (1–2)	105 (56.8)
	Poor (3–5)	54 (29.2)
“B” symptoms (%)	Yes	41 (22.2)
	No	139 (75.1)
	Missing	5 (2.7)
Bulky disease (%)	Yes	58 (31.4)
	No	122 (65.9)
	Missing	5 (2.7)
Bone marrow involvement (%)	Yes	23 (12.4)
	No	140 (75.7)
	Missing	22 (11.9)
Performance status (ECOG) (%)	0	115 (62.2)
	1	58 (31.4)
	2	12 (6.5)

## Data Availability

Array data are available on the NCBI GEO repository as GSE186441.

## Supplementary Materials

The following supporting information can be downloaded at: https://www.mdpi.com/article/10.3390/cancers15102753/s1. Refs. [67,68,69] are cited in Supplementary Materials.
