# Supplementary material for: Exploratory Genome-Wide Association Analysis to Identify Pharmacogenetic Determinants of Response to R-CHOP in Diffuse Large B-Cell Lymphoma
Source: Cancers (Basel). 2023 May 13;15(10):2753. doi: 10.3390/cancers15102753 (PMC10216814; doi:10.3390/cancers15102753)
Supplement: Supplementary file 1 [file cancers-15-02753-s001.zip › cancers-2345720-supplementary.pdf]

Methods

1. Quality control process

Sample quality metrics. To evaluate the samples quality, the following sample quality control metrics were used:

1. DQC (Dish Quality Control): it is based on intensities of probe sequences for non-polymorphic genome sites and measures the resolution of contrast values using AT signal and GC signal: $Contrast \approx \frac{ATSignal-GCSignal}{ATSignal+GCSignal}$. DQC near to 1 means a high quality sample and DQC near to zero means a low quality samples. DQC threshold is 0.82. [67];
2. QCCR (Quality Control Call Rate): it is the proportion of genotyped SNPs for a sample. QCCR threshold is 97% [67];
3. PPR (Plate Pass Rate): it measures the quality of the plate, and it is the proportion of samples on the plate passing the previous two filters [67];
4. APCR (Average Plate Call Rate): it is the mean of the quality control call rate of samples passing the DQC and QCCR filters [67].

Plates can be classified as “Passing-quality” if APCR ≥ 98.5% or as “High-quality” if PPR ≥ 95% and APCR ≥ 99%.

SNP quality metrics. To evaluate the SNP quality, we used some metrics of which the most important are:

1. CR (Call Rate): it is the proportion of genotyped samples for a specific SNP [67];
2. FLD (Fisher's Linear Discriminant): it measures the cluster quality of a SNP considering the distance between the cluster centers in the X dimension. FLD is the smallest distance between the heterozygous cluster center and the two homozygous cluster centers. High FLD means well-separated cluster, thus high quality [67];
3. HetSO (Heterozygous Strength Offset): it measures the distance of cluster centers in the Y dimension. High HetSO value means high quality [67];
4. HomRO (Homozygous Ratio Offset): it measures the distance in X dimension of the homozygous cluster centers from zero. Approximately the heterozygous center should be zero, the AA cluster should be on the right side of zero and the BB cluster on the left side of zero. A negative value of HomRO means that there is a homozygous cluster on the wrong side [67].

These metrics do not filter out directly the SNPs; they are needed to classify the SNPs into the following groups: PolyHighResolution, MonoHighResolution, NoMinorHom, CallRateBelowThreshold, OTV and Hemizygous [67].

To filter the SNPs, the following thresholds were used:

1. BestProbeset = 1: some probesets were linked to the same SNP; in this way a unique probeset corresponded to each SNP;
2. BestandRecommended = 1: only SNPs classified as PolyHighResolution, NoMinorHom, MonoHighResolution and Hemizygous were considered since they were the recommended groups;
3. ConversionType ≠MonoHighResolution: monomorphic SNPs were removed;
4. CR ≥ 95%: SNPs with a low call rate were removed;
5. To get SNPs with good cluster properties the following thresholds were applied:

5.1. FLD ≥ 3.6;
5.2. HetSo ≥ -0.1;
5.3. HomRo1 ≥ 0.6 (for SNPs with 1 cluster);
5.4. HomRo2 ≥ 0.3 (for SNPs with 2 clusters);
5.5. HomRo3 ≥ -0.9 (for SNPs with 3 clusters);

6. H.W.pvalue > $1e^{-10}$: it is the significance threshold for the Hardy-Weinberg equilibrium test;
7. MAF > 0.01

After applying these filters, 633290 out of 86727 SNPs remained, and they were considered in the association analysis.

Sex check: Differences between the annotated sex and genotyped sex were evaluated using intensities and heterozygosity rates of chromosomes X and Y.

Figures

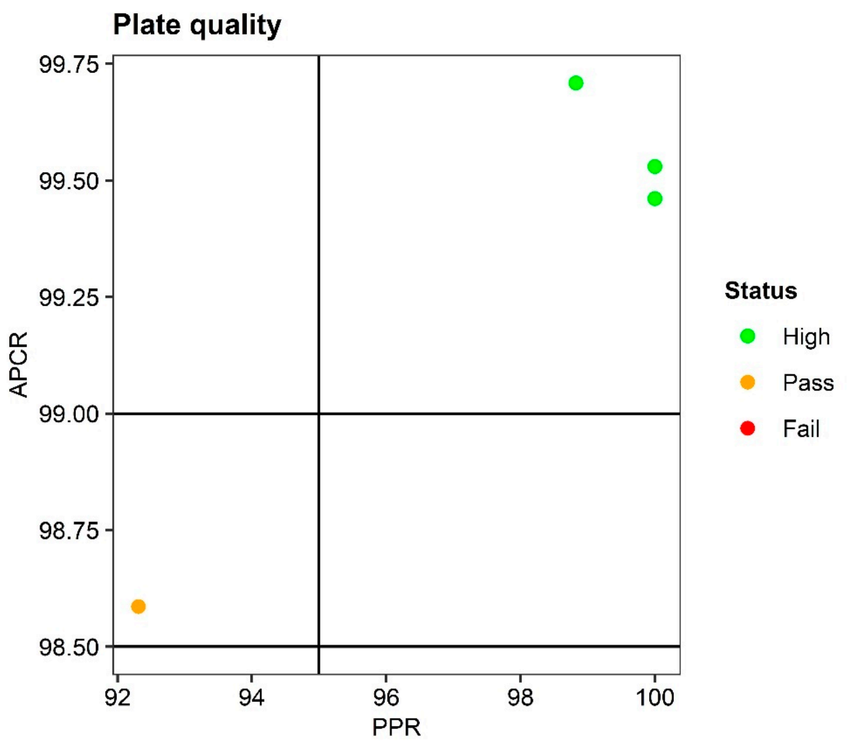

Supplemental Figure S1: Plate quality metrics. On the horizontal axis the PPR and on the vertical axis the APCR are reported. Green points represent High-quality plates, yellow points Passing-quality plates and red ones are plates that do not achieve the quality thresholds indicated by the two horizontal lines at 98.5 and 99 for APCR and by the vertical line at 95 for PPR. From top to bottom: plate 1, 4,3 and 2. All plates reached the thresholds: only plate 2 had a Passing-quality, the others had a High-quality.

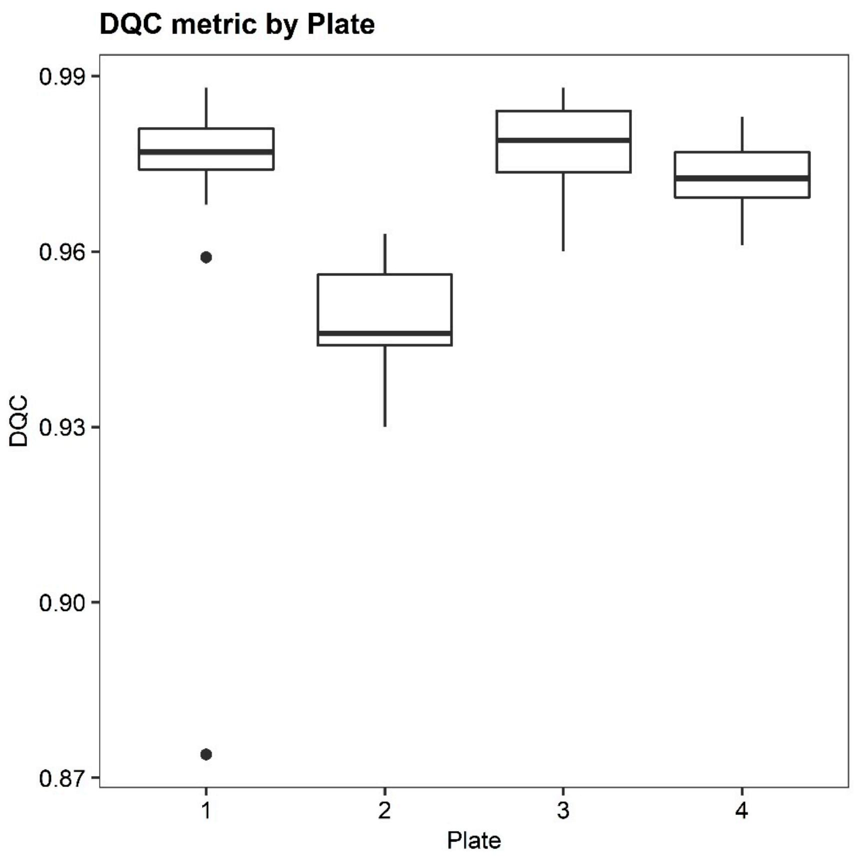

Supplemental Figure S2: Summary of DQC metric across the plates. Plate 2 had the lowest DQC average (0.95). Sample with the lowest DQC (0.87) was in plate 1. All the samples achieved the DQC threshold 0.82.

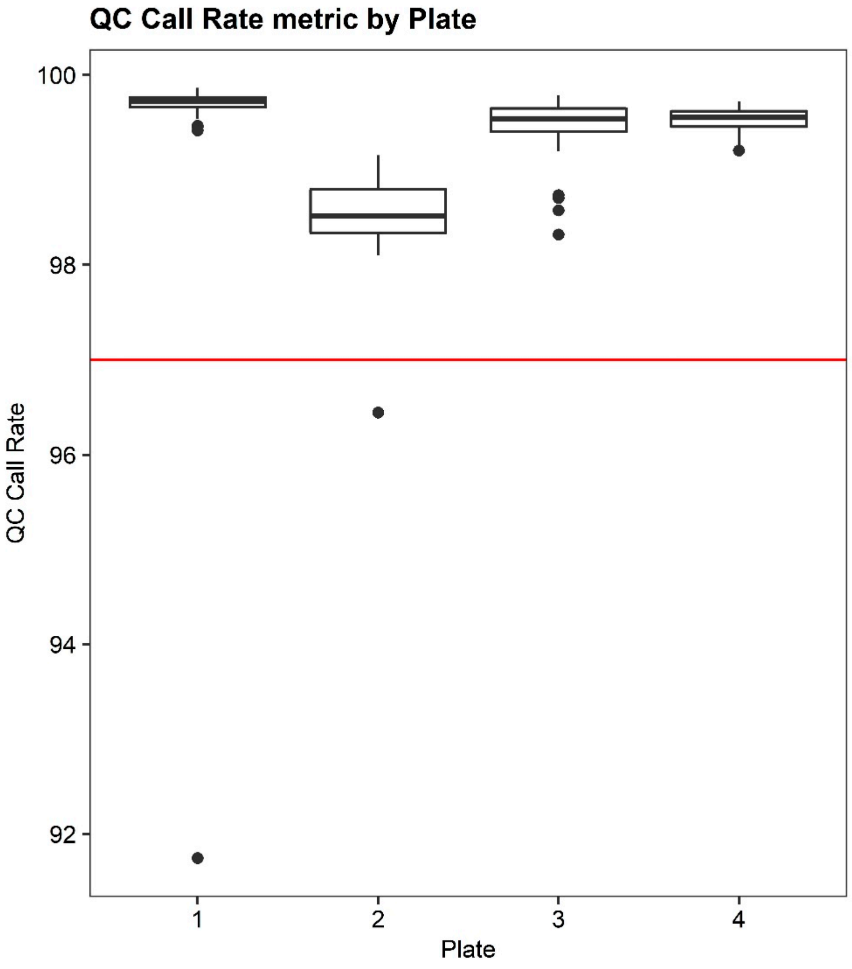

Supplemental Figure S3: Summary of QCCR metric across the plates. The horizontal red line represents the QCCR thresholds (97%). Two samples did not reach the threshold: one in plate 1 (91.75%) and one in plate 2 (96.45%). Plate 2 had the lowest QCCR average (98.4%).

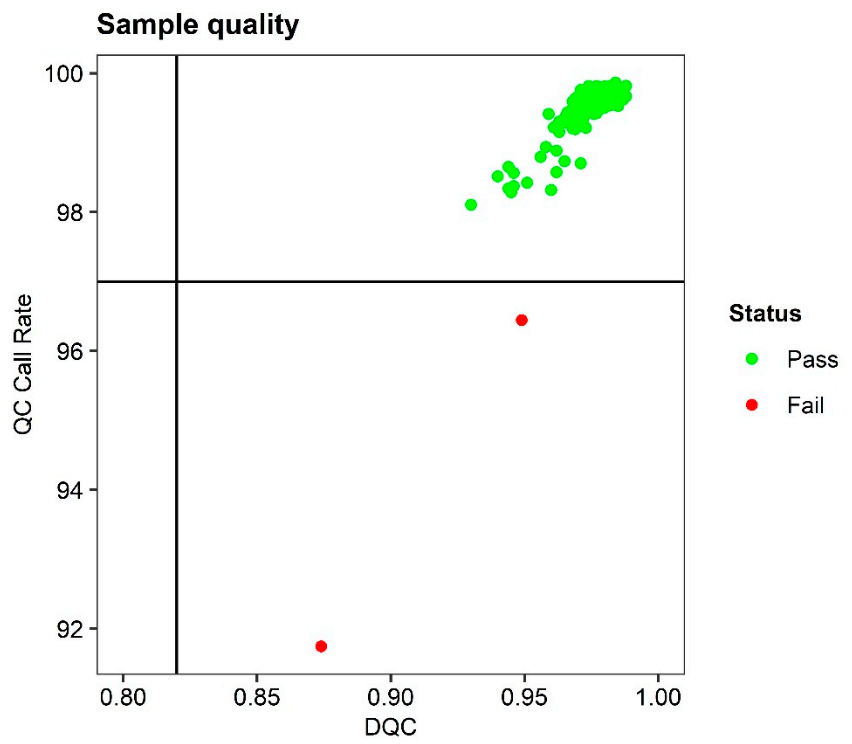

Supplemental Figure S4: Sample quality metrics. On horizontal axis the DQC and on vertical axis the QCCR. The vertical line at 0.82 and the horizontal line at 97% represent the thresholds of DQC and QCCR respectively. Sample in the top-right rectangle represented by green points passed both quality metrics; samples in bottom-right rectangle failed the QCCR. Failed samples were not genotyped.

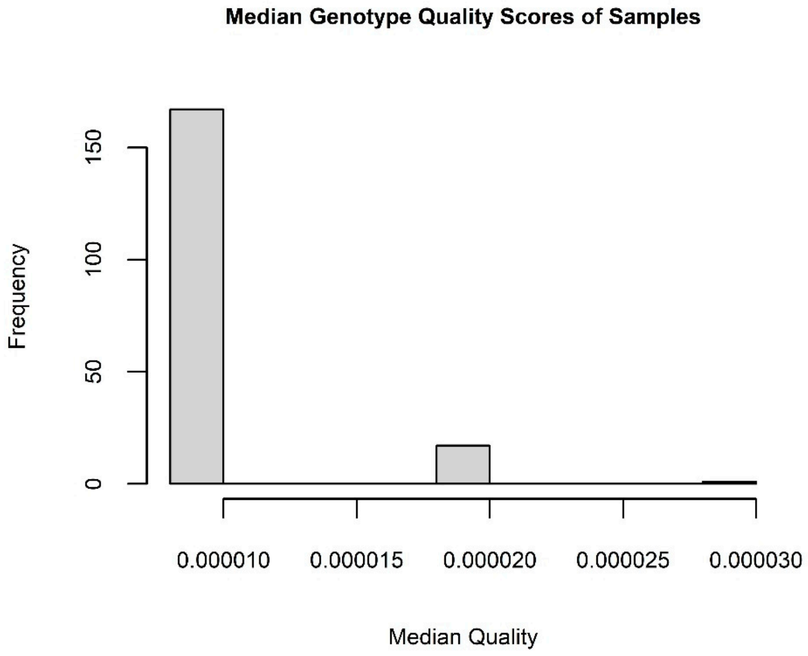

Supplemental Figure S5: Frequency of the median confidence score (CS) per sample. The median CS ranges in between 10^{-5} and $3x10^{-5}$, thus samples had median high quality calls according to CS.

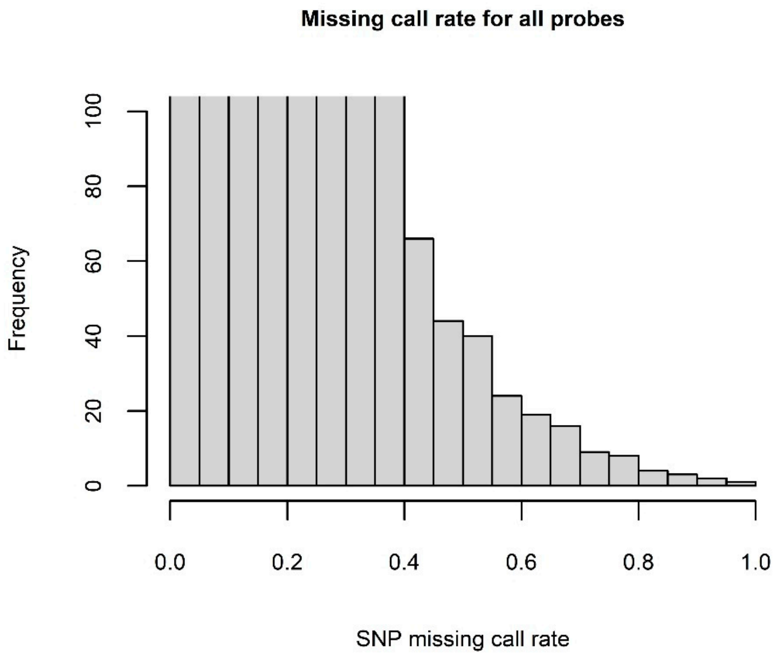

Supplemental Figure S6: Frequencies of SNP missing call rates for all probes [68].

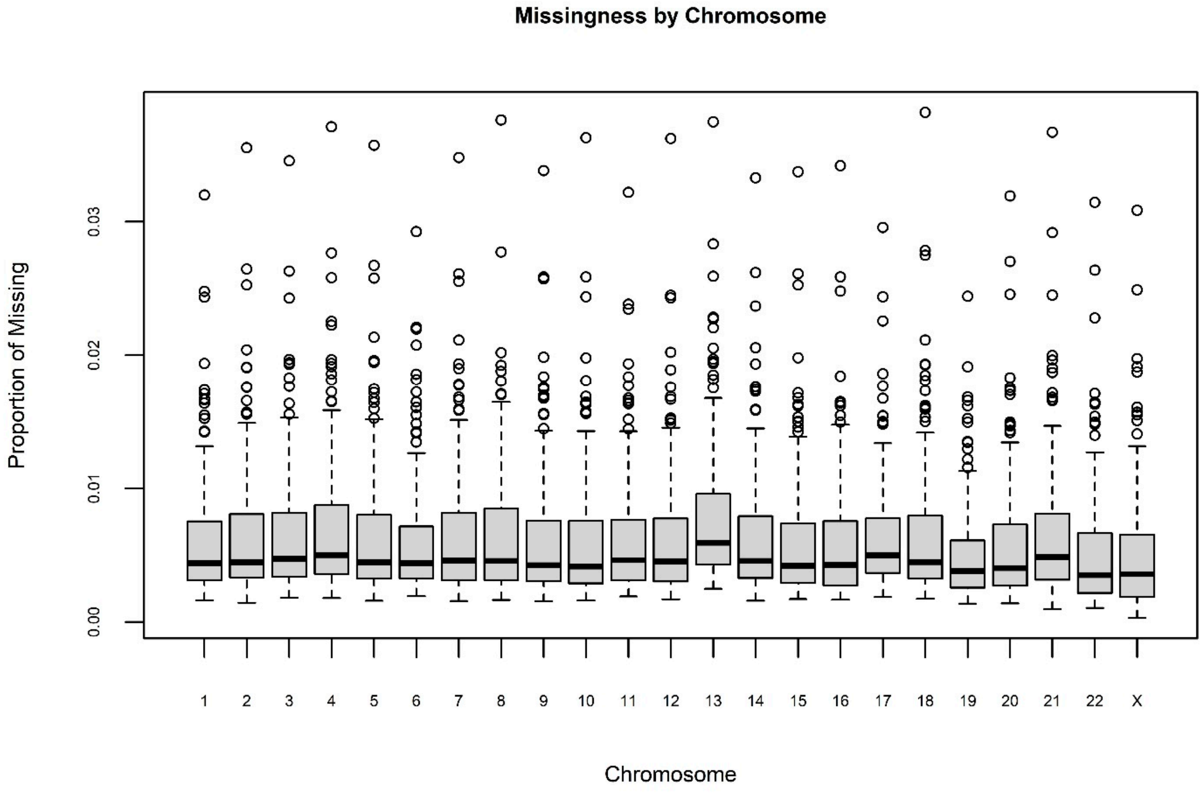

Supplemental Figure S7: Summary of SNP missing call rate for each chromosome. Chromosome 13 had the highest average of missing values [68].

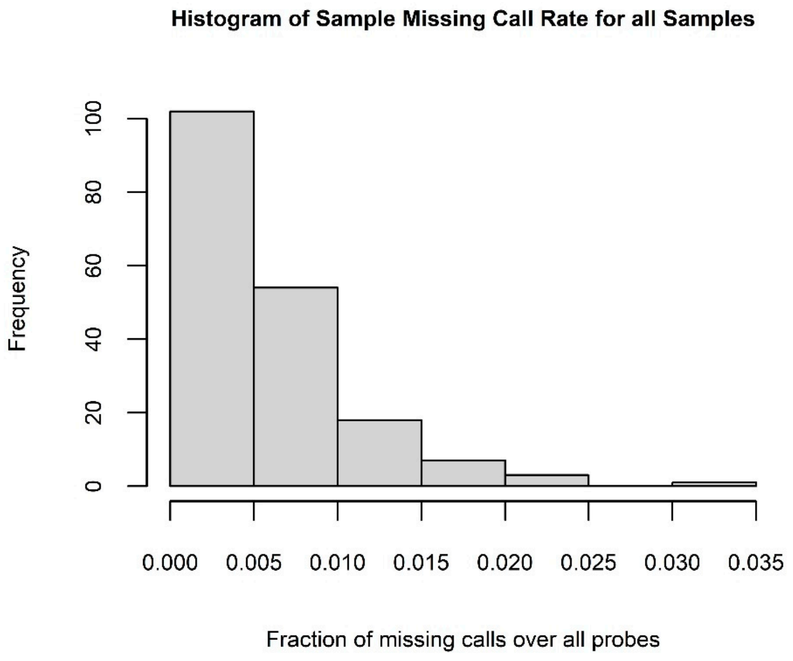

Supplemental Figure S8: Frequency of missing call rates for all 185 samples [68].

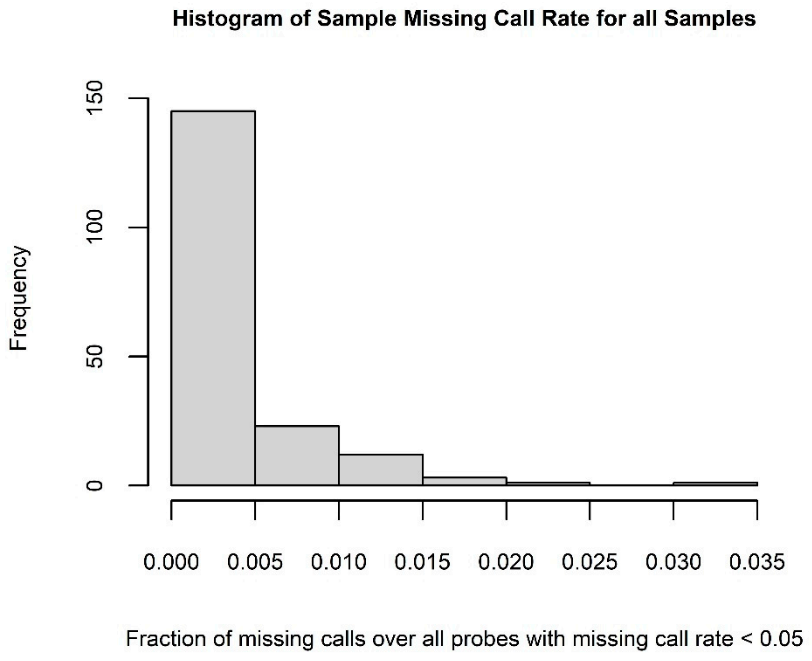

Supplemental Figure S9: Frequency of missing call rates for probes with a call rate below the 0.05 threshold [68].

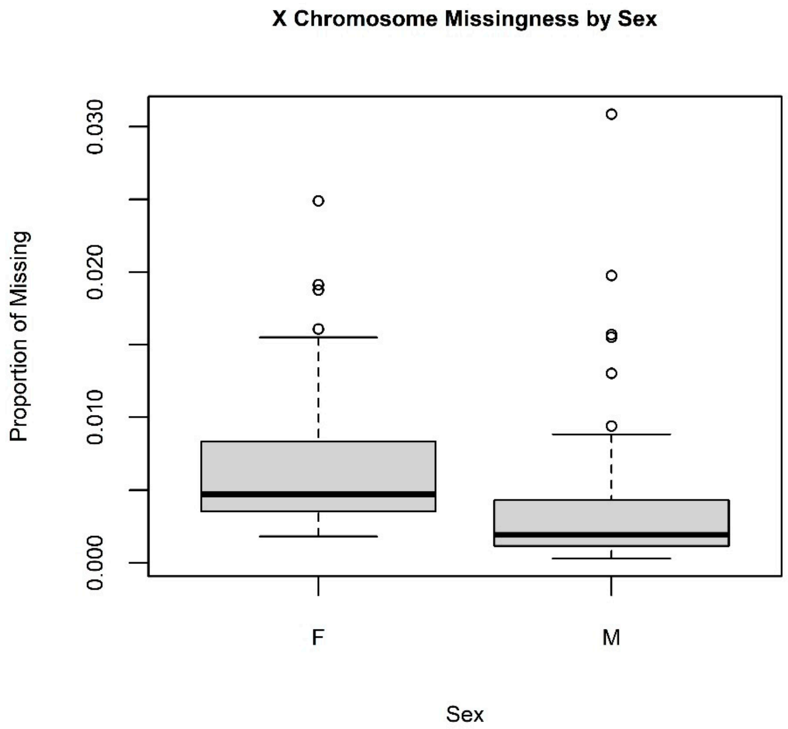

Supplemental Figure S10: Summary of missing call rate on chromosome X by sex [68].

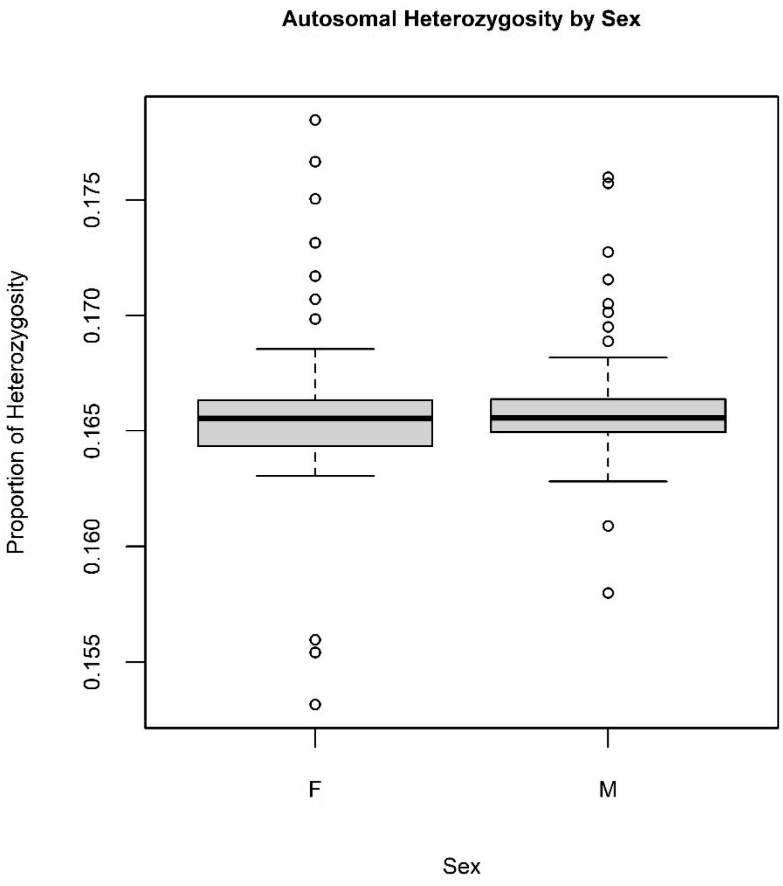

Supplemental Figure S11: Summary of heterozygosity on autosomal chromosomes by sex [68].

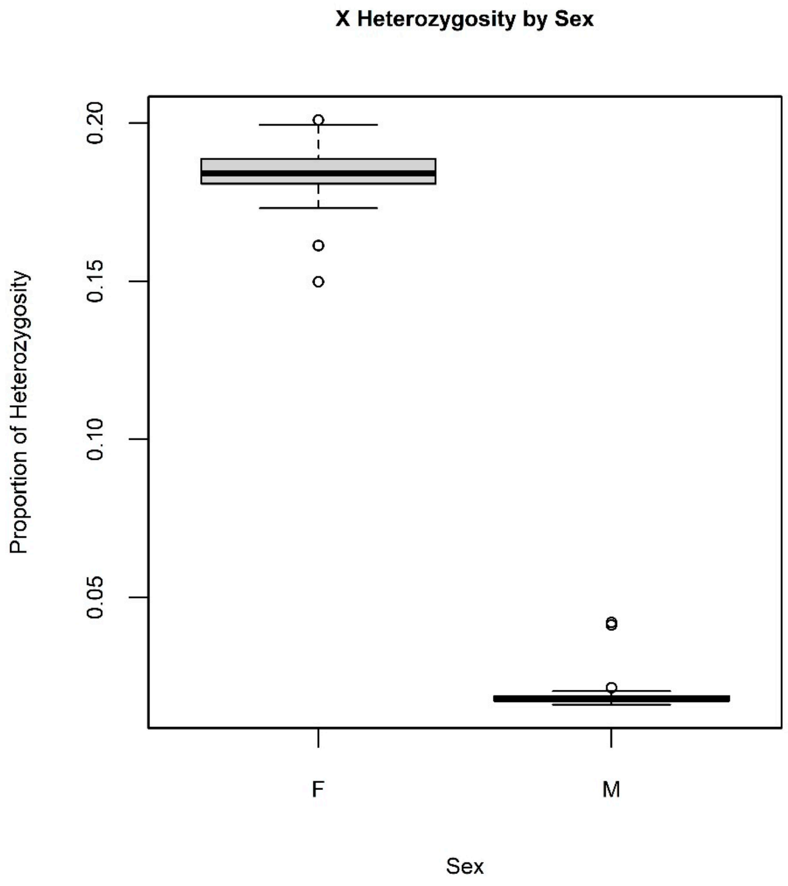

Supplemental Figure S12: Summary of heterozygosity on chromosome X by sex [68].

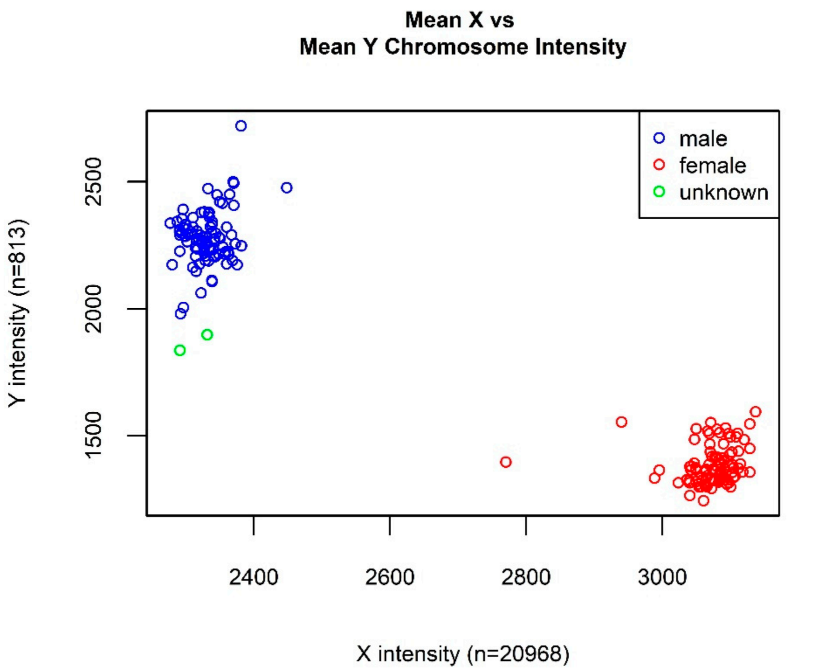

Supplemental Figure S13: X vs Y intensity. Blue points represent genotyped male samples, red points genotyped female samples and the green ones samples whose sex was not genotyped. The closeness of green points to the male cluster confirmed the male sex reported in our database [68],[69].

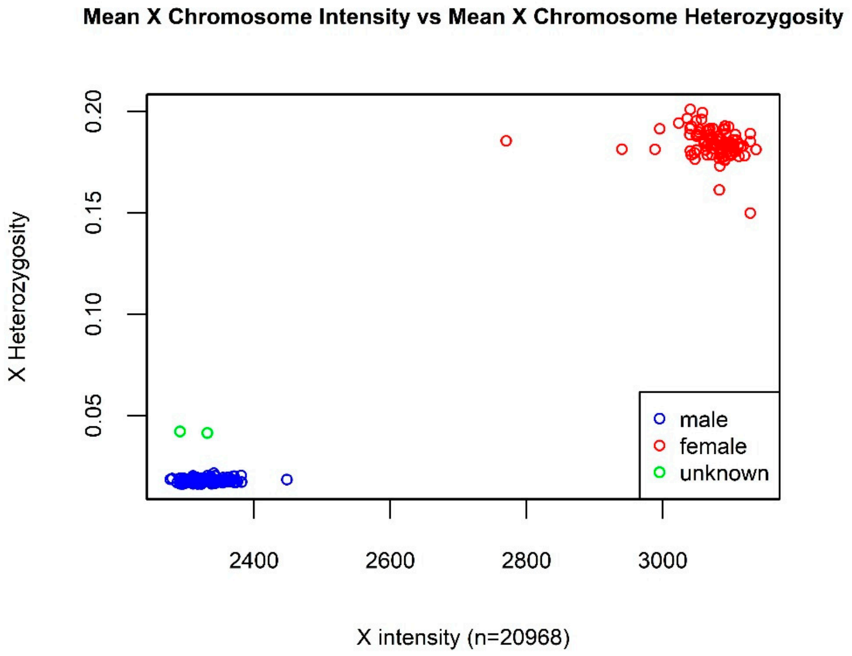

Supplemental Figure S14: X intensity vs X heterozigosity. Blue points represent genotyped male samples, red points genotyped female samples and the green ones samples whose sex was not genotyped. The closeness of green points to the male cluster confirmed the male sex reported in our database [68],[690].

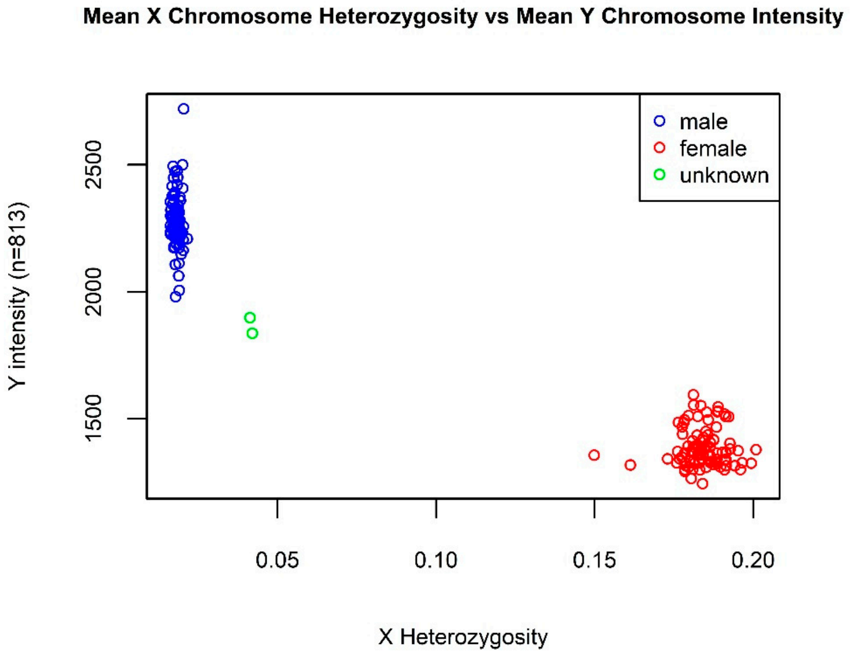

Supplemental Figure S15: X heterozygosity vs Y intensity. Blue points represent genotyped male samples, red points genotyped female samples and the green ones samples whose sex was not genotyped. The closeness of green points to the male cluster confirmed the male sex reported in our database [68],[69].

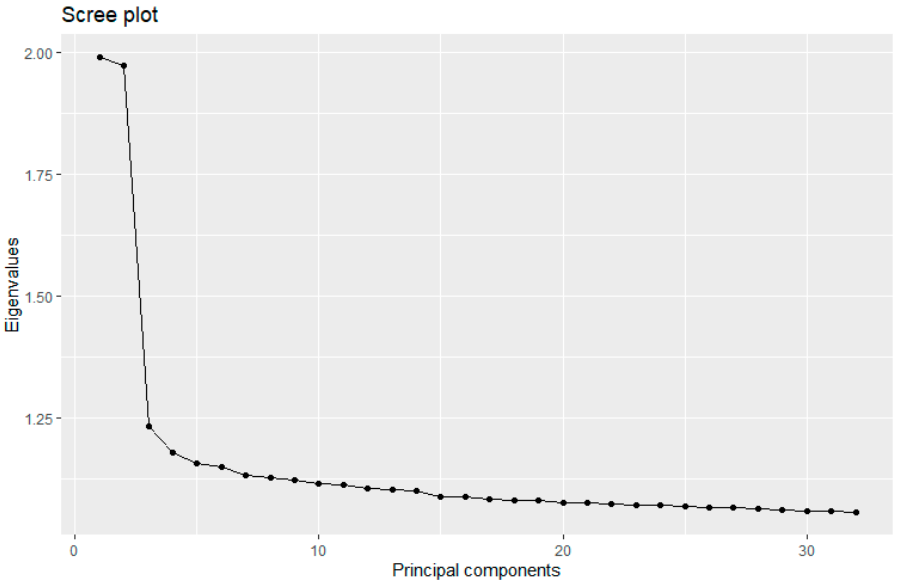

Supplemental Figure S16: Scree-plot of principal components analysis

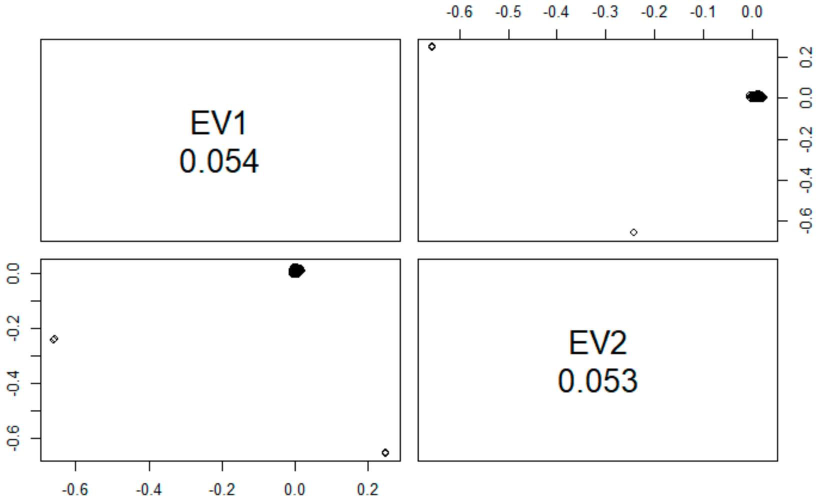

Supplemental Figure S17: Eigenvectors of the first two principal components

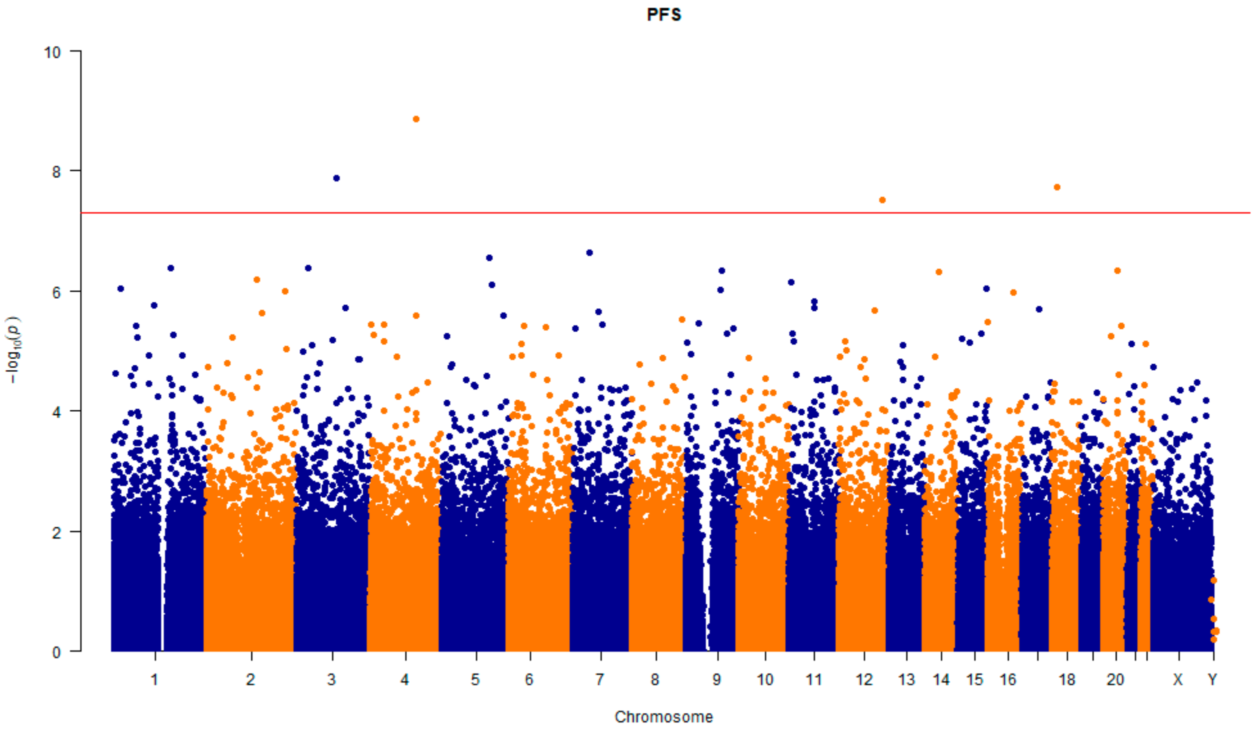

Supplemental Figure S18: Manhattan plot generated from the results of the association test (univariate Cox regression) between Progression Free Survival (PFS) and the SNPs. On the horizontal axis chromosomes from 1 to 22 followed by X, Y and mitochondrial DNA (M) and on the vertical axis the log-transformed p-values of the associations. The grey dashed horizontal line represents the GWAS threshold for significance $5x10^{-8}$. Four SNPs achieved the threshold [68].

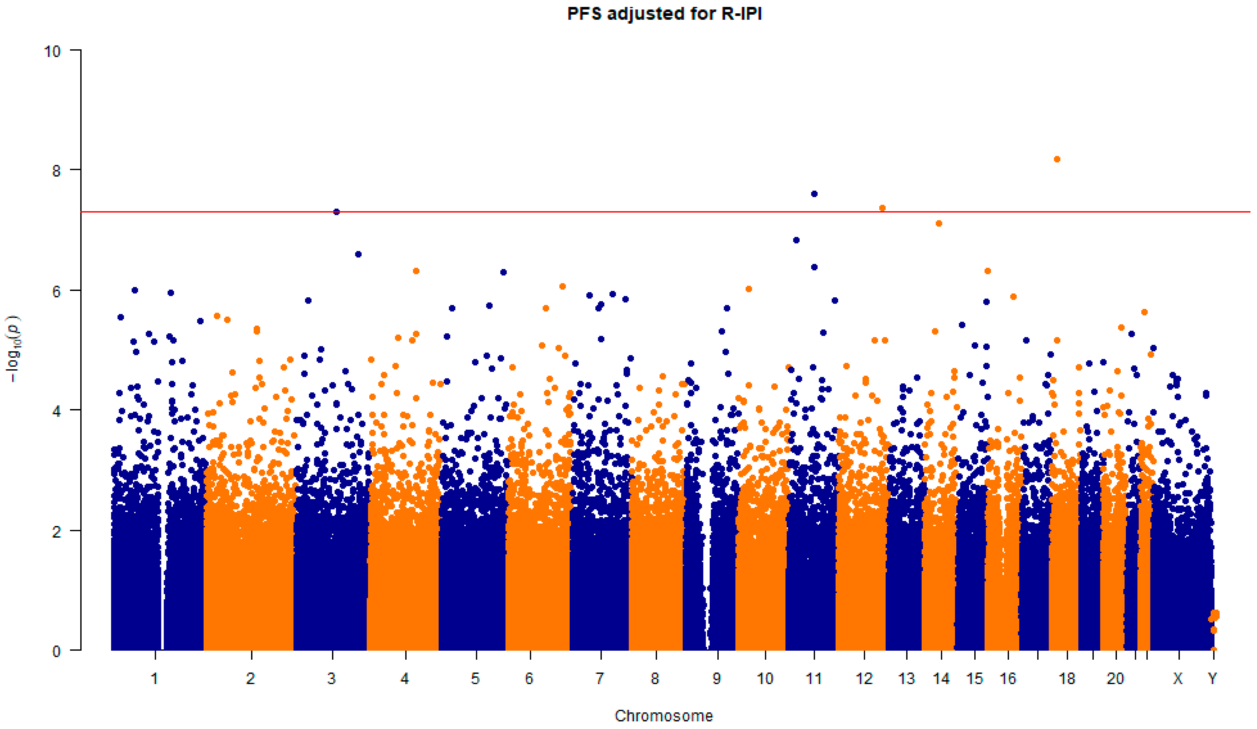

Supplemental Figure S19: Manhattan plot generated from the results of the association test (univariate Cox regression) between Progression Free Survival (PFS) and the SNPs adjusted for the Revised International Prognostic Index (R-IPI). On the horizontal axis chromosomes from 1 to 22 followed by X, Y and mitochondrial DNA (M) and on the vertical axis the log-transformed p-values of the associations. The orange horizontal line represents the GWAS threshold for significance $5x10^{-8}$. Three SNPs achieved the threshold [68].

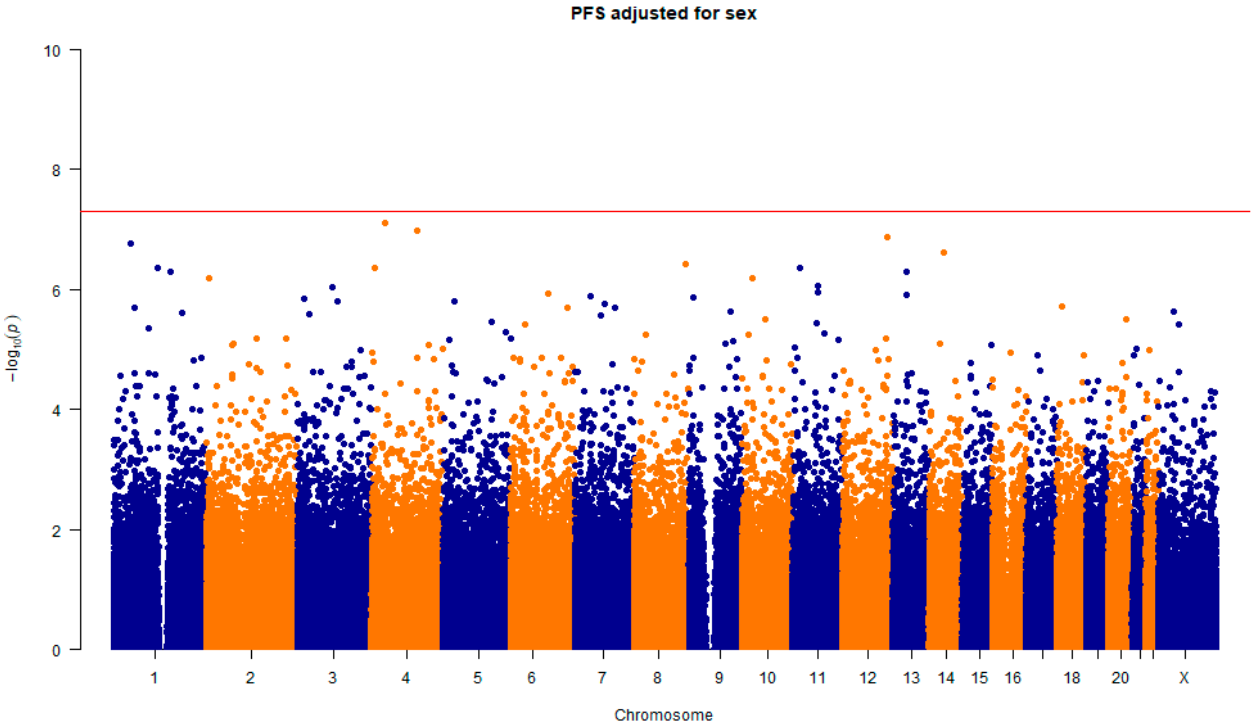

Supplemental Figure S20: Manhattan plot generated from the results of the association test (univariate Cox

regression) between the Progression Free Survival (PFS) and the SNPs adjusted for sex. On the horizontal axis chromosomes from 1 to 22 followed by X and mitochondrial DNA (M) and on the vertical axis the log-transformed p-values of the associations. The orange horizontal line represents the GWAS threshold for significance 5x10^{-8}. No SNPs achieved the threshold [68].

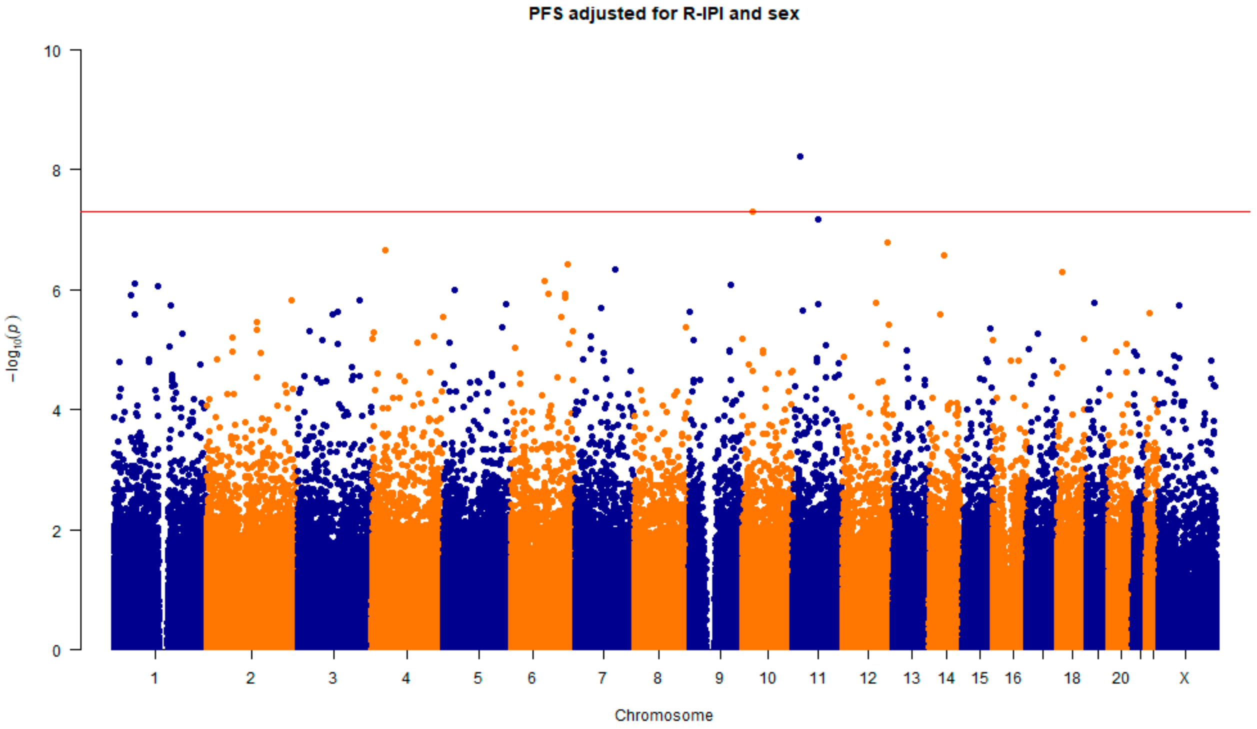

Supplemental Figure S21: Manhattan plot generated from the results of the association test (univariate Cox regression) between the Progression Free Survival (PFS) and the SNPs adjusted for R-IPI and sex. On the horizontal axis chromosomes from 1 to 22 followed by X and mitochondrial DNA (M) and on the vertical axis the log-transformed p-values of the associations. The orange horizontal line represents the GWAS threshold for significance 5x10^{-8}. One SNP achieved the threshold [68].

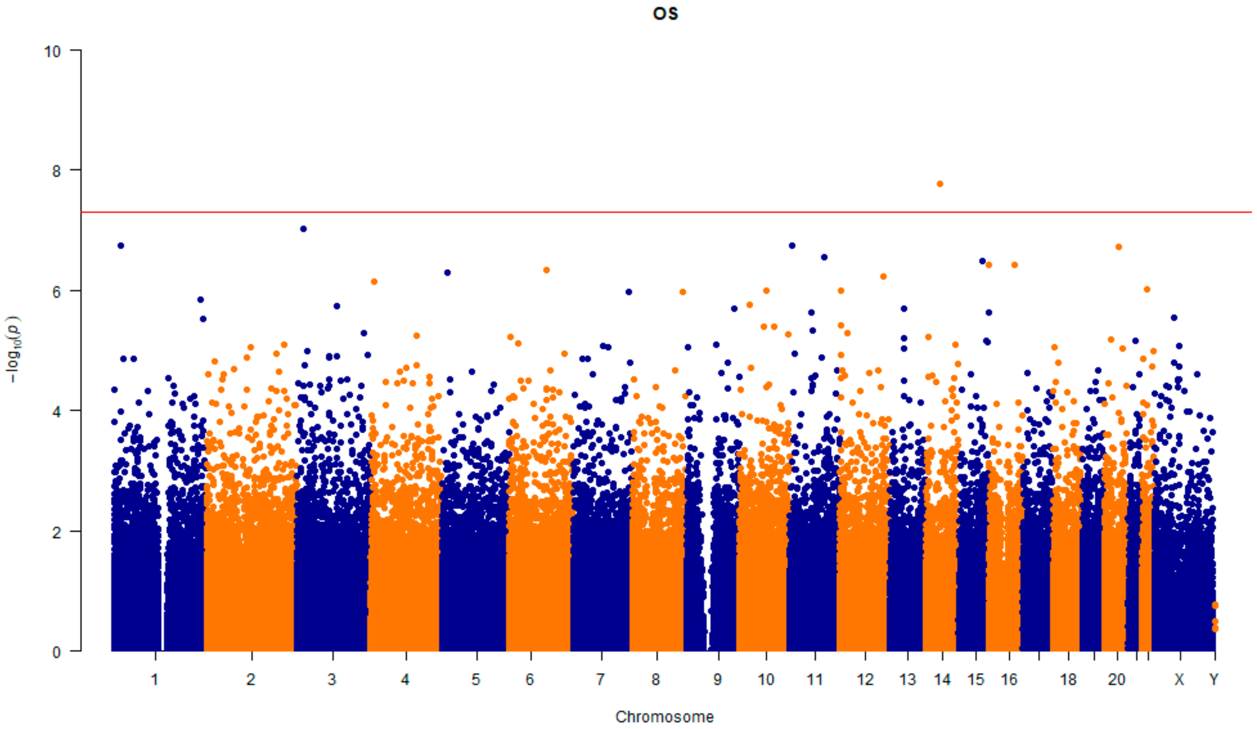

Supplemental Figure S23: Manhattan plot generated from the results of the association test (univariate Cox regression) between the Overall Survival (OS) and the SNPs adjusted for sex. On the horizontal axis chromosomes from 1 to 22 followed by X and mitochondrial DNA (M) and on the vertical axis the log-transformed p-values of the associations. The orange horizontal line represents the GWAS threshold for significance 5x10^{-8}. One SNP achieved the threshold [68].

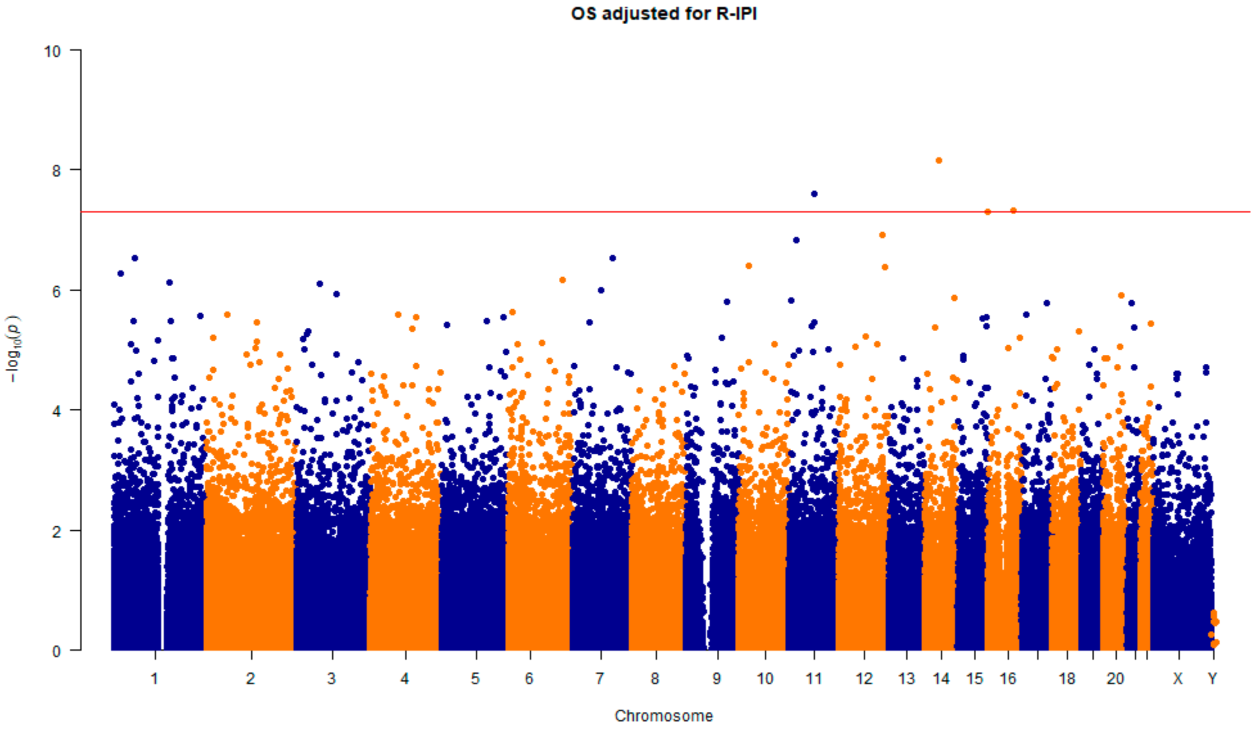

Supplemental Figure S22: Manhattan plot generated from the results of the association test (univariate Cox regression) between the Overall Survival (OS) and the SNPs adjusted for the Revised International Prognostic Index (R-IPI). On the horizontal axis chromosomes from 1 to 22 followed by X, Y and mitochondrial DNA (M) and on the vertical axis the log-transformed p-values of the associations. The orange horizontal line represents the GWAS threshold for significance $5x10^{-8}$. Three SNPs achieved the threshold [68].

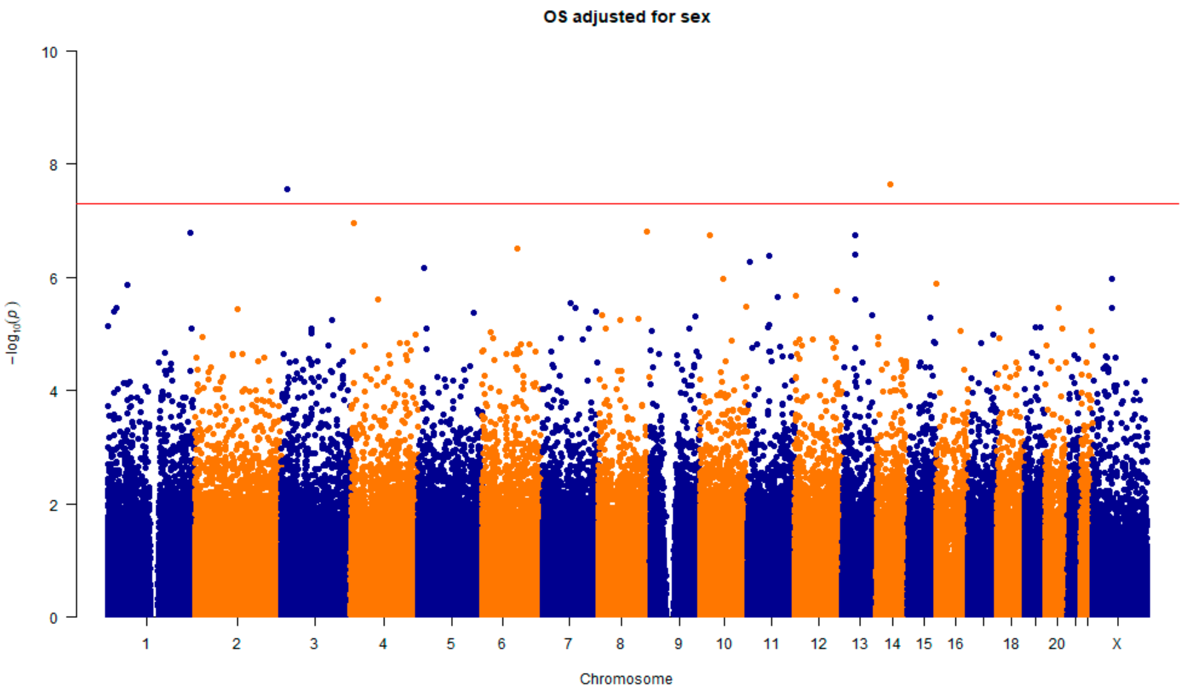

Supplemental Figure S24: Manhattan plot generated from the results of the association test (univariate Cox regression) between the Overall Survival (OS) and the SNPs adjusted for sex. On the horizontal axis chromosomes from 1 to 22 followed by X and mitochondrial DNA (M) and on the vertical axis the log-transformed p-values of the associations. The orange horizontal line represents the GWAS threshold for significance $5x10^{-8}$. Two SNPs achieved the threshold [68].

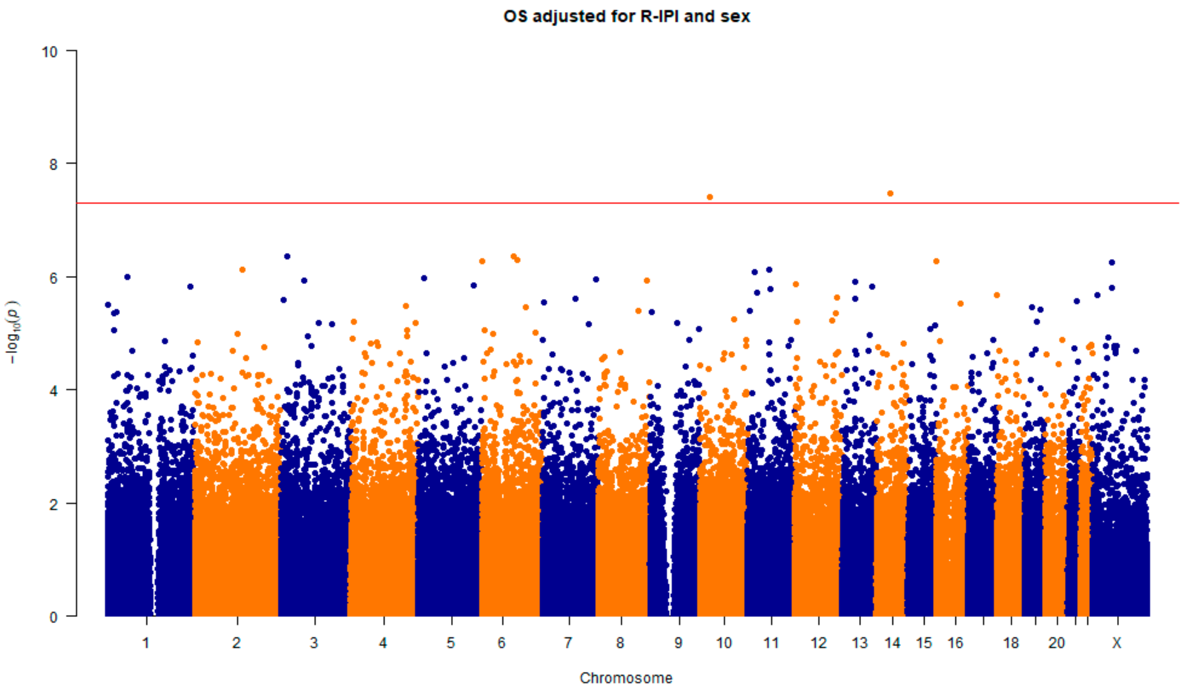

Supplemental Figure S25: Manhattan plot generated from the results of the association test (univariate Cox regression) between the Overall Survival (OS) and the SNPs adjusted for R-IPI and sex. On the horizontal axis chromosomes from 1 to 22 followed by X and mitochondrial DNA (M) and on the vertical axis the log-transformed p-values of the associations. The orange horizontal line represents the GWAS threshold for significance $5x10^{-8}$. Two SNPs achieved the threshold [68].

Tables

Supplemental tables from 1 to 8 report the results of preliminary association tests and association tests adjusted for R-IPI, sex and both R-IPI and sex.

For progression free survival (PFS) and overall survival (OS) univariate Cox regressions were performed. Tables report snpID, rsID, chr (chromosome), n (number of samples), events (number of samples with progression), HR (hazard ratio), p (p-value), MAF (minor allele frequency), Alleles (reference/alternate allele) and the gene. Results are reported in order of significance until 10^{-6}.

Supplemental tables from 9 to 24 report the results association between the five kind of toxicity (neutropenia, haematological toxicity, gastrointestinal toxicity, infection and maximum toxicity) and SNPs or clinical/pathological characteristics of the patients. Univariate logistic regressions were performed (both G0 vs G1-4 and G0-2 vs G3-4); for each model OR (odds ratio), CI (confidence interval) and p (p-value) are reported.

Results of gastrointestinal toxicity and infection G0-2 vs G3-4 are not reported because of the imbalance between the two groups: only 2 and 4 samples were in the G3-4 group for gastrointestinal toxicity and infection respectively.

Supplemental Table S1. PFS

snpID	rsID	chr	pos	n	n.events	HR	p	MAF	Allele.A	Allele.B	effect.allele	gene
214862	rs116665727	4	122085516	184	39	6.77	1e-09	0.03	C	G	B	TNIP3
159779	rs1607795	3	106521561	184	39	18.56	1.3e-08	0.01	T	C	A	—
722955	rs75614943	18	12273488	185	39	15.73	1.9e-08	0.02	T	C	B	CIDEA
569870	rs77241831	12	117763282	185	39	6.66	3.1e-08	0.05	A	G	A	NOS1
350689	rs217416	7	44564735	182	39	3.07	2.31e-07	0.23	T	C	B	NPC1L1
263789	rs13169004	5	129793171	185	39	8.14	2.78e-07	0.03	T	C	A	—
141218	rs138677120	3	32586521	181	39	10.16	4.16e-07	0.02	T	C	A	DYNC1LI1
37209	rs116114495	1	154924015	185	39	4.85	4.22e-07	0.05	T	C	A	PBXIP1
440029	rs117408675	9	96487128	185	39	7.83	4.62e-07	0.03	T	C	A	PHF2
779956	rs117914915	20	37684359	183	39	15.29	4.65e-07	0.01	T	G	A	LOC339568
616310	rs74832512	14	58451211	185	39	15.67	4.86e-07	0.01	T	C	A	SLC35F4
101083	rs7582472	2	134644794	185	39	3.30	6.42e-07	0.26	T	C	B	—
498257	rs117433832	11	5651500	185	39	23.88	7.26e-07	0.01	A	G	A	TRIM34
265189	rs78508722	5	135631174	185	39	14.70	8.13e-07	0.01	A	G	A	TRPC7
7337	rs77034259	1	18213429	185	39	7.17	9.26e-07	0.01	A	C	A	ACTL8
655204	rs77417635	15	99157593	185	39	7.98	9.48e-07	0.02	T	C	B	FAM169B
439118	rs117559338	9	93106201	182	39	13.94	9.52e-07	0.02	A	G	A	LOC101927873

Supplemental Table S2. PFS adjusted for sex

snpID	rsID	chr	pos	n	n.events	HR	p	MAF	Allele.A	Allele.B	effect.allele	gene
195588	rs16992033	4	36284941	184	39	9.66	8e-08	0.03	T	C	B	DTHD1
214862	rs116665727	4	122085516	184	39	5.53	1.06e-07	0.03	C	G	B	TNIP3
569870	rs77241831	12	117763282	185	39	6.11	1.37e-07	0.05	A	G	A	NOS1
16601	rs72681903	1	47132446	185	39	21.01	1.76e-07	0.02	A	G	A	ATPAF1
616310	rs74832512	14	58451211	185	39	19.45	2.49e-07	0.01	T	C	A	SLC35F4
415959	rs118148193	8	136101301	185	39	18.66	3.76e-07	0.01	T	C	B	—
34538	rs115467558	1	120163899	185	39	16.00	4.4e-07	0.02	A	G	B	ZNF697
34542	rs114915501	1	120170684	185	39	16.00	4.4e-07	0.02	T	C	A	ZNF697
503450	rs78466241	11	19284671	185	39	15.00	4.44e-07	0.02	C	G	B	—
187960	rs76342242	4	7818811	185	39	17.21	4.5e-07	0.01	A	G	A	AFAP1
589418	rs117143161	13	56252083	184	39	5.98	5.04e-07	0.05	A	G	A	—
37209	rs116114495	1	154924015	185	39	5.14	5.23e-07	0.05	T	C	A	PBXIP1
465381	rs12356569	10	29405545	184	39	17.50	6.63e-07	0.01	T	G	A	LYZL1
67974	rs114125706	2	6319471	185	39	12.30	6.7e-07	0.02	T	C	A	—
517406	rs117500207	11	68362253	184	39	13.16	8.69e-07	0.02	T	C	B	PPP6R3
157485	rs9878351	3	95075462	185	39	4.75	9.49e-07	0.08	A	C	A	—

Supplemental Table S3. PFS adjusted for R-IPI

snpID	rsID	chr	pos	n	n.events	HR	p	MAF	Allele.A	Allele.B	effect.allele	gene
722955	rs75614943	18	12273488	185	39	20.67	7e-09	0.02	T	C	B	CIDEA
517406	rs117500207	11	68362253	184	39	24.38	2.6e-08	0.02	T	C	B	PPP6R3
569870	rs77241831	12	117763282	185	39	6.58	4.5e-08	0.05	A	G	A	NOS1
159779	rs1607795	3	106521561	184	39	15.98	5e-08	0.01	T	C	A	—
616310	rs74832512	14	58451211	185	39	30.69	7.9e-08	0.01	T	C	A	SLC35F4
503450	rs78466241	11	19284671	185	39	16.82	1.51e-07	0.02	C	G	B	—
174144	rs116598269	3	166990117	184	39	15.60	2.54e-07	0.02	T	C	A	ZBBX
517208	rs113862077	11	67727252	185	39	13.04	4.16e-07	0.02	T	C	B	—
214862	rs116665727	4	122085516	184	39	5.28	4.89e-07	0.03	C	G	B	TNIP3
656821	rs61759901	16	320992	185	39	28.89	4.89e-07	0.01	A	C	A	RGS11
274190	rs12655922	5	166999625	185	39	9.04	5.22e-07	0.02	A	T	A	TENM2
327186	rs117818775	6	144798077	184	39	18.27	8.88e-07	0.01	C	G	A	UTRN

Supplemental Table S3. PFS adjusted for R-IPI

snpID	rsID	chr	pos	n	n.events	HR	p	MAF	Allele.A	Allele.B	effect.allele	gene
327193		6	144831624	183	39	18.36	8.99e-07	0.01	A	C	B	UTRN
465381	rs12356569	10	29405545	184	39	16.55	9.68e-07	0.01	T	G	A	LYZL1

Supplemental Table S4. PFS adjusted for sex and R-IPI

snpID	rsID	chr	pos	n	n.events	HR	p	MAF	Allele.A	Allele.B	effect.allele	gene
503450	rs78466241	11	19284671	185	39	32.42	6e-09	0.02	C	G	B	—
465381	rs12356569	10	29405545	184	39	27.64	5.1e-08	0.01	T	G	A	LYZL1
517406	rs117500207	11	68362253	184	39	22.08	6.8e-08	0.02	T	C	B	PPP6R3
569870	rs77241831	12	117763282	185	39	6.08	1.67e-07	0.05	A	G	A	NOS1
195588	rs16992033	4	36284941	184	39	9.71	2.26e-07	0.03	T	C	B	DTHD1
616310	rs74832512	14	58451211	185	39	25.87	2.67e-07	0.01	T	C	A	SLC35F4
329589	rs13203258	6	152691271	185	39	12.57	3.87e-07	0.02	T	C	A	SYNE1
364999	rs4386902	7	107873120	185	39	6.50	4.64e-07	0.03	T	C	B	NRCAM
722955	rs75614943	18	12273488	185	39	14.75	5.03e-07	0.02	T	C	B	CIDEA
314026	rs117534791	6	90599072	185	39	40.76	7.15e-07	0.01	T	C	A	GJA10
18705	rs114412656	1	55718442	185	39	29.13	8.01e-07	0.01	T	C	A	—
444941	rs117013765	9	113254657	184	39	18.70	8.38e-07	0.01	A	G	B	SVEP1
34538	rs115467558	1	120163899	185	39	14.26	8.67e-07	0.02	A	G	B	ZNF697
34542	rs114915501	1	120170684	185	39	14.26	8.67e-07	0.02	T	C	A	ZNF697

Supplemental Table S5. OS

snpID	rsID	chr	pos	n	n.events	HR	p	MAF	Allele.A	Allele.B	effect.allele	gene
616310	rs74832512	14	58451211	185	26	26.48	1.7e-08	0.01	T	C	A	SLC35F4
136710	rs11721010	3	16348323	185	26	15.05	9.6e-08	0.02	T	C	B	OXNAD1
498257	rs117433832	11	5651500	185	26	38.97	1.84e-07	0.01	A	G	A	TRIM34
7337	rs77034259	1	18213429	185	26	9.06	1.85e-07	0.01	A	C	A	ACTL8
779956	rs117914915	20	37684359	183	26	18.20	1.9e-07	0.01	T	G	A	LOC339568
524069	rs72972650	11	93364390	185	26	11.09	2.82e-07	0.02	T	C	A	KIAA1731
649715	rs72746927	15	84443493	185	26	13.54	3.25e-07	0.03	A	G	A	ADAMTSL3

Supplemental Table S5. OS

snpID	rsID	chr	pos	n	n.events	HR	p	MAF	Allele.A	Allele.B	effect.allele	gene
676781	rs35789195	16	68730515	185	26	9.71	3.75e-07	0.03	A	G	A	CDH3
656821	rs61759901	16	320992	185	26	36.27	3.82e-07	0.01	A	C	A	RGS11
316409	rs79497891	6	101376121	185	26	16.09	4.68e-07	0.01	A	C	A	—
238441	rs74795805	5	15896558	185	26	29.83	5.21e-07	0.01	A	C	A	FBXL7
569870	rs77241831	12	117763282	185	26	7.57	5.96e-07	0.05	A	G	A	NOS1
187960	rs76342242	4	7818811	185	26	16.75	7.13e-07	0.01	A	G	A	AFAP1
806586	rs73162123	22	32081043	185	26	15.30	9.67e-07	0.01	T	C	B	PRR14L

Supplemental Table S6. OS adjusted for sex

snpID	rsID	chr	pos	n	n.events	HR	p	MAF	Allele.A	Allele.B	effect.allele	gene
616310	rs74832512	14	58451211	185	26	30.63	2.3e-08	0.01	T	C	A	SLC35F4
136710	rs11721010	3	16348323	185	26	20.30	2.7e-08	0.02	T	C	B	OXNAD1
187960	rs76342242	4	7818811	185	26	25.35	1.11e-07	0.01	A	G	A	AFAP1
415959	rs118148193	8	136101301	185	26	22.22	1.59e-07	0.01	T	C	B	—
60062	rs4330980	1	234098310	185	26	9.15	1.67e-07	0.04	A	G	A	SLC35F3
465381	rs12356569	10	29405545	184	26	24.57	1.83e-07	0.01	T	G	A	LYZL1
589418	rs117143161	13	56252083	184	26	6.93	1.84e-07	0.05	A	G	A	—
316409	rs79497891	6	101376121	185	26	18.13	3.19e-07	0.01	A	C	A	—
589437	rs117754604	13	56358817	184	26	6.75	4.07e-07	0.05	A	C	B	—
514367	rs76689084	11	60914411	184	26	8.83	4.29e-07	0.05	T	C	A	VPS37C
498257	rs117433832	11	5651500	185	26	33.55	5.47e-07	0.01	A	G	A	TRIM34
238441	rs74795805	5	15896558	185	26	30.38	6.79e-07	0.01	A	C	A	FBXL7

Supplemental Table S7. OS adjusted for R-IPI

snpID	rsID	chr	pos	n	n.events	HR	p	MAF	Allele.A	Allele.B	effect.allele	gene
616310	rs74832512	14	58451211	185	39	56.03	7e-09	0.01	T	C	A	SLC35F4
517406	rs117500207	11	68362253	184	39	25.46	2.6e-08	0.02	T	C	B	PPP6R3
676781	rs35789195	16	68730515	185	39	9.65	4.9e-08	0.03	A	G	A	CDH3
656821	rs61759901	16	320992	185	39	57.30	5.1e-08	0.01	A	C	A	RGS11
569870	rs77241831	12	117763282	185	39	6.19	1.24e-07	0.05	A	G	A	NOS1
503450	rs78466241	11	19284671	185	39	19.73	1.49e-07	0.02	C	G	B	—

Supplemental Table S7. OS adjusted for R-IPI

snpID	rsID	chr	pos	n	n.events	HR	p	MAF	Allele.A	Allele.B	effect.allele	gene
364999	rs4386902	7	107873120	185	39	6.39	2.98e-07	0.03	T	C	B	NRCAM
19272	rs72672663	1	57628145	184	39	5.68	3.01e-07	0.09	T	C	B	DAB1
465381	rs12356569	10	29405545	184	39	19.38	4.11e-07	0.01	T	G	A	LYZL1
571576	rs35933842	12	121570899	184	39	17.81	4.22e-07	0.01	T	G	A	P2RX7
7337	rs77034259	1	18213429	185	39	11.42	5.52e-07	0.01	A	C	A	ACTL8
327193		6	144831624	183	39	19.65	6.81e-07	0.01	A	C	B	UTRN
327186	rs117818775	6	144798077	184	39	19.49	7.04e-07	0.01	C	G	A	UTRN
35808	rs78426848	1	150984035	184	39	10.93	7.6e-07	0.02	T	G	A	PRUNE
150869	rs35971523	3	62301277	183	39	12.29	8.1e-07	0.02	T	C	A	PTPRG-AS1

Supplemental Table S8. OS adjusted for sex and R-IPI

snpID	rsID	chr	pos	n	n.events	HR	p	MAF	Allele.A	Allele.B	effect.allele	gene
616310	rs74832512	14	58451211	185	26	53.91	3.4e-08	0.01	T	C	A	SLC35F4
465381	rs12356569	10	29405545	184	26	39.70	4e-08	0.01	T	G	A	LYZL1
314026	rs117534791	6	90599072	185	26	52.02	4.34e-07	0.01	T	C	A	GJA10
136710	rs11721010	3	16348323	185	26	16.18	4.38e-07	0.02	T	C	B	OXNAD1
316409	rs79497891	6	101376121	185	26	17.09	5.24e-07	0.01	A	C	A	—
279854	rs114755190	6	856884	185	26	12.83	5.41e-07	0.03	T	C	A	LOC101930081
656821	rs61759901	16	320992	185	26	45.15	5.5e-07	0.01	A	C	A	RGS11
823383	rs73208426	23	53056885	185	26	7.80	5.61e-07	0.03	T	C	A	GPR173
514367	rs76689084	11	60914411	184	26	8.84	7.59e-07	0.05	T	C	A	VPS37C
100609	rs7561046	2	132621207	182	26	33.75	7.75e-07	0.01	T	C	B	—
503450	rs78466241	11	19284671	185	26	30.30	8.2e-07	0.02	C	G	B	—

Supplemental Table S9. Maximum toxicity G0 vs G1-4

snpID	rsID	chr	pos	G0	G1-4	OR	p	MAF	Allele.A	Allele.B	effect.allele	gene
610030	rs8005039	14	33269788	36	147	0.28	1.57e-05	0.34	A	G	B	AKAP6
232391	rs464466	4	189543953	34	143	0.15	2.13e-05	0.09	A	G	B	LINC01060
542687	rs79539049	12	12728951	35	149	0.07	2.61e-05	0.04	A	C	B	—
480873	rs74150005	10	91600399	36	149	0.19	3.79e-05	0.10	A	C	A	LINC00865
388403	rs77893236	8	18913647	36	149	0.23	4.37e-05	0.12	A	G	A	PSD3
152750	rs115176051	3	68397633	36	149	0.06	4.80e-05	0.04	A	G	B	FAM19A1
480877	rs10509582	10	91617124	34	149	0.18	5.75e-05	0.09	A	G	B	LINC01375
361626	rs10280571	7	96986462	36	149	0.17	6.50e-05	0.08	A	G	B	—
401198	rs62518395	8	73136907	36	147	0.21	6.61e-05	0.11	T	C	B	—
728266	rs34112764	18	37549233	35	149	0.11	6.91e-05	0.05	A	G	B	LOC101927900
484076	rs66765139	10	103015827	35	147	0.29	7.17e-05	0.21	A	G	B	LOC101927419
419660	rs7854418	9	335545	35	148	0.30	7.87e-05	0.36	A	G	B	DOCK8
130196	NA	3	1562218	35	149	0.09	8.32e-05	0.04	T	G	B	—
560021	rs76319852	12	80987678	36	149	0.17	8.34e-05	0.07	A	G	A	—
603211	rs9577826	13	112584379	36	149	0.30	9.64e-05	0.27	A	T	A	—

Supplemental Table S10. Maximum toxicity G0 vs G1-4 adjusted for sex

snpID	rsID	chr	pos	G0	G1-4	OR	p	MAF	Allele.A	Allele.B	effect.allele	gene
232391	rs464466	4	189543953	34	143	0.15	1.74e-05	0.09	A	G	B	LINC01060
603211	rs9577826	13	112584379	36	149	0.23	1.77e-05	0.27	A	T	A	—
610030	rs8005039	14	33269788	36	147	0.29	3.36e-05	0.34	A	G	B	AKAP6
152750	rs115176051	3	68397633	36	149	0.05	4.21e-05	0.04	A	G	B	FAM19A1
273893	rs74612488	5	165950937	36	149	0.18	4.90e-05	0.08	A	G	A	—

Supplemental Table S10. Maximum toxicity G0 vs G1-4 adjusted for sex

snpID	rsID	chr	pos	G0	G1-4	OR	p	MAF	Allele.A	Allele.B	effect.allele	gene
363074	rs4727484	7	100928622	36	149	0.30	5.67e-05	0.29	A	G	A	—
542687	rs79539049	12	12728951	35	149	0.07	5.87e-05	0.04	A	C	B	—
388403	rs77893236	8	18913647	36	149	0.24	6.22e-05	0.12	A	G	A	PSD3
401198	rs62518395	8	73136907	36	147	0.20	6.89e-05	0.11	T	C	B	—
560021	rs76319852	12	80987678	36	149	0.16	6.91e-05	0.07	A	G	A	—
480873	rs74150005	10	91600399	36	149	0.20	8.63e-05	0.10	A	C	A	LINC00865
612796	rs12147387	14	44921353	36	149	0.17	8.64e-05	0.09	C	G	B	—
635287	rs4924541	15	35397948	36	147	0.25	8.90e-05	0.46	T	C	A	—
419660	rs7854418	9	335545	35	148	0.30	9.61e-05	0.36	A	G	B	DOCK8
137671	rs115416707	3	20887917	36	148	0.09	9.65e-05	0.04	A	G	B	—
85760	rs10496105	2	64320746	36	149	0.23	9.76e-05	0.14	A	G	A	PELI1

Supplemental Table S11. Maximum toxicity G0 vs G1-4 adjusted for R-IPI

snpID	rsID	chr	pos	G0	G1-4	OR	p	MAF	Allele.A	Allele.B	effect.allele	gene
610030	rs8005039	14	33269788	36	147	0.28	1.53e-05	0.34	A	G	B	AKAP6
542687	rs79539049	12	12728951	35	149	0.06	1.94e-05	0.04	A	C	B	—
232391	rs464466	4	189543953	34	143	0.15	2.34e-05	0.09	A	G	B	LINC01060
388403	rs77893236	8	18913647	36	149	0.21	2.59e-05	0.12	A	G	A	PSD3
480873	rs74150005	10	91600399	36	149	0.19	3.94e-05	0.10	A	C	A	LINC00865
152750	rs115176051	3	68397633	36	149	0.06	5.44e-05	0.04	A	G	B	FAM19A1
728266	rs34112764	18	37549233	35	149	0.10	5.72e-05	0.05	A	G	B	LOC101927900
130196	NA	3	1562218	35	149	0.08	6.39e-05	0.04	T	G	B	—
480877	rs10509582	10	91617124	34	149	0.18	7.25e-05	0.09	A	G	B	LINC01375
560021	rs76319852	12	80987678	36	149	0.17	7.81e-05	0.07	A	G	A	—
401198	rs62518395	8	73136907	36	147	0.21	8.14e-05	0.11	T	C	B	—

Supplemental Table S11. Maximum toxicity G0 vs G1-4 adjusted for R-IPI

snpID	rsID	chr	pos	G0	G1-4	OR	p	MAF	Allele.A	Allele.B	effect.allele	gene
484076	rs66765139	10	103015827	35	147	0.29	8.73e-05	0.21	A	G	B	LOC101927419
419660	rs7854418	9	335545	35	148	0.29	8.81e-05	0.36	A	G	B	DOCK8
699521	rs8067577	17	35088881	35	145	3.99	8.89e-05	0.44	A	G	A	—
361626	rs10280571	7	96986462	36	149	0.17	9.43e-05	0.08	A	G	B	—
120793	rs111533869	2	218655603	36	149	0.16	9.71e-05	0.06	C	G	A	TNS1

Supplemental Table S12. Maximum toxicity G0 vs G1-4 adjusted for sex and R-IPI

snpID	rsID	chr	pos	G0	G1-4	OR	p	MAF	Allele.A	Allele.B	effect.allele	gene
232391	rs464466	4	189543953	34	143	0.15	1.98e-05	0.09	A	G	B	LINC01060
603211	rs9577826	13	112584379	36	149	0.24	2.43e-05	0.27	A	T	A	—
610030	rs8005039	14	33269788	36	147	0.28	2.94e-05	0.34	A	G	B	AKAP6
388403	rs77893236	8	18913647	36	149	0.21	3.46e-05	0.12	A	G	A	PSD3
542687	rs79539049	12	12728951	35	149	0.07	4.43e-05	0.04	A	C	B	—
152750	rs115176051	3	68397633	36	149	0.05	5.50e-05	0.04	A	G	B	FAM19A1
363074	rs4727484	7	100928622	36	149	0.29	5.59e-05	0.29	A	G	A	—
560021	rs76319852	12	80987678	36	149	0.16	6.81e-05	0.07	A	G	A	—
273893	rs74612488	5	165950937	36	149	0.18	7.21e-05	0.08	A	G	A	—
127491	rs1874573	2	238301184	36	147	0.28	7.71e-05	0.31	A	G	B	COL6A3
130196	NA	3	1562218	35	149	0.08	7.99e-05	0.04	T	G	B	—
249452	rs3730089	5	67588148	36	149	0.22	8.10e-05	0.16	A	G	A	PIK3R1
401198	rs62518395	8	73136907	36	147	0.20	8.36e-05	0.11	T	C	B	—

Supplemental Table S12. Maximum toxicity G0 vs G1-4 adjusted for sex and R-IPI

snpID	rsID	chr	pos	G0	G1-4	OR	p	MAF	Allele.A	Allele.B	effect.allele	gene
453318	rs4841953	9	137904288	36	149	0.29	8.92e-05	0.36	A	C	B	OLFM1
728266	rs34112764	18	37549233	35	149	0.11	9.10e-05	0.05	A	G	B	LOC101927900
480873	rs74150005	10	91600399	36	149	0.20	9.68e-05	0.10	A	C	A	LINC00865
699521	rs8067577	17	35088881	35	145	4.15	9.68e-05	0.44	A	G	A	—
85760	rs10496105	2	64320746	36	149	0.22	9.82e-05	0.14	A	G	A	PELI1
635287	rs4924541	15	35397948	36	147	0.25	9.84e-05	0.46	T	C	A	—

Supplemental Table S13. Maximum toxicity G0-2 vs G3-4

snpID	rsID	chr	pos	G0-2	G3-4	OR	p	MAF	Allele.A	Allele.B	effect.allele	gene
165026	NA	3	127253736	115	70	3.79	3.60e-06	0.25	A	G	A	LOC101927149
482131	rs2860840	10	96495232	115	70	2.91	5.95e-05	0.28	T	C	A	CYP2C18
77280	rs7589917	2	36615654	114	70	0.34	6.68e-05	0.34	A	G	B	CRIM1
307889	rs77450031	6	57080434	115	70	7.23	6.78e-05	0.07	C	G	A	RAB23
245779	rs9647518	5	51720930	115	70	2.52	6.88e-05	0.49	A	G	B	—
219144	rs3796700	4	141787969	113	69	0.33	7.88e-05	0.33	T	C	A	RNF150
403217	rs2912803	8	82595445	115	70	4.06	8.14e-05	0.12	A	G	A	IMPA1
219122	rs4535404	4	141723078	114	70	2.72	8.68e-05	0.29	A	G	A	—
83005	rs13402444	2	51534381	114	70	2.82	8.81e-05	0.28	T	C	B	LOC730100
109645	rs7585843	2	171385679	114	70	0.34	9.16e-05	0.29	A	G	B	MYO3B

Supplemental Table S14. Maximum toxicity G0-2 vs G3-4 adjusted for sex

snpID	rsID	chr	pos	G0-2	G3-4	OR	p	MAF	Allele.A	Allele.B	effect.allele	gene
165026	NA	3	127253736	115	70	3.77	3.90e-06	0.25	A	G	A	LOC101927149
245779	rs9647518	5	51720930	115	70	2.59	5.25e-05	0.49	A	G	B	—
482131	rs2860840	10	96495232	115	70	2.92	5.40e-05	0.28	T	C	A	CYP2C18
109645	rs7585843	2	171385679	114	70	0.32	6.33e-05	0.29	A	G	B	MYO3B
307889	rs77450031	6	57080434	115	70	7.28	6.59e-05	0.07	C	G	A	RAB23
83005	rs13402444	2	51534381	114	70	2.88	7.25e-05	0.28	T	C	B	LOC730100
77280	rs7589917	2	36615654	114	70	0.33	8.62e-05	0.34	A	G	B	CRIM1
219144	rs3796700	4	141787969	113	69	0.33	8.66e-05	0.33	T	C	A	RNF150
219122	rs4535404	4	141723078	114	70	2.73	8.72e-05	0.29	A	G	A	—
403217	rs2912803	8	82595445	115	70	4.02	9.20e-05	0.12	A	G	A	IMPA1
358125	rs66694457	7	80971638	115	70	3.24	9.41e-05	0.18	A	G	B	—

Supplemental Table S15. Maximum toxicity G0-2 vs G3-4 adjusted for R-IPI

snpID	rsID	chr	pos	G0-2	G3-4	OR	p	MAF	Allele.A	Allele.B	effect.allele	gene
165026	NA	3	127253736	115	70	3.69	7.00e-06	0.25	A	G	A	LOC101927149
77280	rs7589917	2	36615654	114	70	0.32	4.82e-05	0.34	A	G	B	CRIM1
482131	rs2860840	10	96495232	115	70	2.98	5.98e-05	0.28	T	C	A	CYP2C18
307889	rs77450031	6	57080434	115	70	7.70	6.37e-05	0.07	C	G	A	RAB23
692060	rs1549331	17	9243072	115	70	3.13	7.84e-05	0.20	T	C	A	STX8
245779	rs9647518	5	51720930	115	70	2.52	7.87e-05	0.49	A	G	B	—
534278	rs1892931	11	126401419	115	70	0.37	8.22e-05	0.38	T	C	B	KIRREL3

Supplemental Table S15. Maximum toxicity G0-2 vs G3-4 adjusted for R-IPI

snpID	rsID	chr	pos	G0-2	G3-4	OR	p	MAF	Allele.A	Allele.B	effect.allele	gene
83005	rs13402444	2	51534381	114	70	2.87	8.41e-05	0.28	T	C	B	LOC730100
219122	rs4535404	4	141723078	114	70	2.78	8.59e-05	0.29	A	G	A	—

Supplemental Table S16. Maximum toxicity G0-2 vs G3-4 adjusted for sex and R-IPI

snpID	rsID	chr	pos	G0-2	G3-4	OR	p	MAF	Allele.A	Allele.B	effect.allele	gene
165026	NA	3	127253736	115	70	3.65	8.50e-06	0.25	A	G	A	LOC101927149
482131	rs2860840	10	96495232	115	70	2.99	5.18e-05	0.28	T	C	A	CYP2C18
68540	rs40998	2	8100132	115	70	7.18	5.65e-05	0.07	C	G	B	LINC00298
534278	rs1892931	11	126401419	115	70	0.36	5.68e-05	0.38	T	C	B	KIRREL3
245779	rs9647518	5	51720930	115	70	2.60	5.68e-05	0.49	A	G	B	—
307889	rs77450031	6	57080434	115	70	7.75	6.32e-05	0.07	C	G	A	RAB23
83005	rs13402444	2	51534381	114	70	2.96	6.47e-05	0.28	T	C	B	LOC730100
77280	rs7589917	2	36615654	114	70	0.32	7.19e-05	0.34	A	G	B	CRIM1
109645	rs7585843	2	171385679	114	70	0.32	7.63e-05	0.29	A	G	B	MYO3B
219122	rs4535404	4	141723078	114	70	2.81	8.84e-05	0.29	A	G	A	—

Supplemental Table S17: Neutropenia G0 vs G1-4

Predictors	OR	CI	p	OR	CI	p	OR	CI	p	OR	CI	p
**Sex: Female**	1.21	0.68 – 2.17	0.511									
**Age**				1.03	1.01 – 1.06	0.004						
**R-IPI: Very good**							0.87	0.35 – 2.07	0.750			
**R-IPI: Bad**							1.73	0.90 – 3.38	0.104			
**Stage: 3-4**										1.58	0.88 – 2.85	0.128
**Observations**	**185**											

Supplemental Table S18: Neutropenia G0-2 vs G3-4

Predictors	OR	CI	p	OR	CI	p	OR	CI	p	OR	CI	p
**Sex: Female**	1.30	0.70 – 2.41	0.405									
**Age**				1.04	1.02 – 1.07	0.002						
**R-IPI: Very good**							0.88	0.32 – 2.23	0.794			
**R-IPI: Bad**							1.77	0.89 – 3.51	0.101			
**Stage: 3-4**										1.45	0.78 – 2.72	0.246
**Observations**	185											

Supplemental Table S19: Haematological toxicity G0 vs G1-4

Predictors	OR	CI	p	OR	CI	p	OR	CI	p	OR	CI	p
**Sex: Female**	1.82	0.99 – 3.36	0.054									
**Age**				1.02	1.00 – 1.04	0.087						
**R-IPI: Very good**							0.95	0.39 – 2.35	0.901			
**R-IPI: Bad**							1.18	0.60 – 2.39	0.635			
**Stage: 3-4**										0.79	0.43 – 1.45	0.457
**Observations**	185											

Supplemental Table S20: Haematological toxicity G0-2 vs G3-4

Predictors	OR	CI	p	OR	CI	p	OR	CI	p	OR	CI	p
**Sex: Female**	1.42	0.78 – 2.61	0.258									
**Age**				1.04	1.02 – 1.07	0.001						
**R-IPI: Very good**							0.80	0.29 – 2.03	0.656			
**R-IPI: Bad**							1.88	0.96 – 3.71	0.067			
**Stage: 3-4**										1.40	0.76 – 2.61	0.278
**Observations**	185											

Supplemental Table S21: Gastrointestinal toxicity G0 vs G1-4

Predictors	OR	CI	p	OR	CI	p	OR	CI	p	OR	CI	p
**Sex: Female**	2.00	0.93 – 4.46	0.081									
**Age**				1.02	0.99 – 1.05	0.175						
**R-IPI: Very good**							1.08	0.33 – 3.05	0.893			
**R-IPI: Bad**							0.91	0.36 – 2.12	0.823			
**Stage: 3-4**										1.16	0.54 – 2.52	0.704
**Observations**	185											

Supplemental Table S22: Infection G0 vs G1-4

Predictors	OR	CI	p	OR	CI	p	OR	CI	p	OR	CI	p
**Sex: Female**	0.83	0.41 – 1.69	0.615									
**Age**				1.01	0.98 – 1.04	0.502						
**R-IPI: Very good**							0.44	0.10 – 1.41	0.211			
**R-IPI: Bad**							0.96	0.43 – 2.09	0.928			
**Stage: 3-4**										0.96	0.47 – 1.97	0.916
**Observations**	**185**											

Supplemental Table S23: Maximum toxicity G0 vs G1-4

Predictors	OR	CI	p	OR	CI	p	OR	CI	p	OR	CI	p
**Sex: Female**	2.00	0.95 – 4.34	0.072									
**Age**				1.01	0.98 – 1.04	0.400						
**R-IPI: Very good**							1.54	0.52 – 5.67	0.464			
**R-IPI: Bad**							1.40	0.61 – 3.43	0.437			
**Stage: 3-4**										0.83	0.39 – 1.72	0.616
**Observations**	**185**											

Supplemental Table S24: Maximum toxicity G0-2 vs G3-4

Predictors	OR	CI	p	OR	CI	p	OR	CI	p	OR	CI	p
**Sex: Female**	1.22	0.67 – 2.22	0.507									
**Age**				1.04	1.02 – 1.07	0.002						
**R-IPI: Very good**							0.65	0.24 – 1.63	0.375			
**R-IPI: Bad**							1.52	0.78 – 2.97	0.218			
**Stage: 3-4**										1.42	0.78 – 2.62	0.250
**Observations**	**185**											

Supplemental Table S25. Objective Response

snpID	rsID	chr	pos	n0	n1	OR	p	MAF	Allele.A	Allele.B	effect.allele	gene
749113	rs72995377	19	1E+07	25	157	0.12	1.56e-05	0.07	A	G	A	ZSWIM4
771636	rs9653638	20	5E+06	26	158	0.21	2.15e-05	0.24	A	G	A	—
730301	rs75215008	18	5E+07	26	158	0.08	3.22e-05	0.04	A	G	A	ZBTB7C
510228	rs4755869	11	4E+07	26	159	0.12	3.71e-05	0.06	A	G	B	—
329319	rs9322337	6	2E+08	26	159	0.11	4.09e-05	0.05	A	C	A	ESR1
189827	rs78741583	4	1E+07	26	159	0.18	4.22e-05	0.14	A	C	B	LINC01085
520052	rs75036351	11	8E+07	25	158	0.05	4.38e-05	0.03	T	C	B	PAK1

428554	rs12552851	9	2E+07	26	159	0.22	4.51e-05	0.18	T	C	B	—
454363	rs118106795	9	1E+08	26	154	0.26	5.44e-05	0.17	A	G	A	CCDC183
756094	rs80203774	19	4E+07	26	159	0.09	5.65e-05	0.04	T	C	A	SIPA1L3
280578	rs4959719	6	3E+06	26	157	0.22	5.99e-05	0.14	T	C	B	MYLK4
164526	rs77302400	3	1E+08	26	159	0.17	6.15e-05	0.07	A	G	B	OSBPL11
351168	rs73112136	7	5E+07	26	157	0.05	6.25e-05	0.03	T	C	A	—
771638	rs6076798	20	5E+06	26	158	0.29	6.52e-05	0.30	A	G	B	—
716116	rs10073	17	8E+07	26	159	6.36	6.85e-05	0.39	T	C	B	ENGASE
314852	rs66522431	6	9E+07	26	159	0.18	7.08e-05	0.14	A	G	A	EPHA7
655371	rs72754844	15	1E+08	26	159	0.19	7.51e-05	0.09	T	C	B	PGPEP1L
722389	rs3748429	18	1E+07	26	159	0.23	8.15e-05	0.16	A	C	B	PIEZO2
722390	rs3748428	18	1E+07	26	159	0.23	8.15e-05	0.16	T	C	A	PIEZO2
410077	rs2068025	8	1E+08	26	159	6.00	9.69e-05	0.39	T	C	A	TRPS1

Supplemental Table S26. Objective Response adjusted for sex

snpID	rsID	chr	pos	n0	n1	OR	p	MAF	Allele.A	Allele.B	effect.allele	gene
749113	rs72995377	19	1E+07	25	157	0.10	1.08e-05	0.07	A	G	A	ZSWIM4
771636	rs9653638	20	5E+06	26	158	0.21	2.21e-05	0.24	A	G	A	—
329319	rs9322337	6	2E+08	26	159	0.09	2.81e-05	0.05	A	C	A	ESR1
510228	rs4755869	11	4E+07	26	159	0.11	3.03e-05	0.06	A	G	B	—
730301	rs75215008	18	5E+07	26	158	0.08	3.37e-05	0.04	A	G	A	ZBTB7C
428554	rs12552851	9	2E+07	26	159	0.21	3.85e-05	0.18	T	C	B	—
756094	rs80203774	19	4E+07	26	159	0.08	4.10e-05	0.04	T	C	A	SIPA1L3
189827	rs78741583	4	1E+07	26	159	0.18	4.63e-05	0.14	A	C	B	LINC01085
520052	rs75036351	11	8E+07	25	158	0.05	5.45e-05	0.03	T	C	B	PAK1
280578	rs4959719	6	3E+06	26	157	0.22	6.12e-05	0.14	T	C	B	MYLK4
314852	rs66522431	6	9E+07	26	159	0.17	6.12e-05	0.14	A	G	A	EPHA7
164526	rs77302400	3	1E+08	26	159	0.17	6.44e-05	0.07	A	G	B	OSBPL11
454363	rs118106795	9	1E+08	26	154	0.26	6.48e-05	0.17	A	G	A	CCDC183
771638	rs6076798	20	5E+06	26	158	0.29	6.56e-05	0.30	A	G	B	—
351168	rs73112136	7	5E+07	26	157	0.05	6.79e-05	0.03	T	C	A	—
722389	rs3748429	18	1E+07	26	159	0.23	7.28e-05	0.16	A	C	B	PIEZO2
722390	rs3748428	18	1E+07	26	159	0.23	7.28e-05	0.16	T	C	A	PIEZO2

716116	rs10073	17	8E+07	26	159	6.32	7.31e-05	0.39	T	C	B	ENGASE
655371	rs72754844	15	1E+08	26	159	0.19	7.49e-05	0.09	T	C	B	PGPEP1L
410077	rs2068025	8	1E+08	26	159	6.04	9.78e-05	0.39	T	C	A	TRPS1

Supplemental Table S27. Objective Response Adjusted for R-IPI

snpID	rsID	chr	pos	n0	n1	OR	p	MAF	Allele.A	Allele.B	effect.allele	gene
771636	rs9653638	20	5E+06	26	158	0.21	3.31e-05	0.24	A	G	A	—
749113	rs72995377	19	1E+07	25	157	0.12	3.53e-05	0.07	A	G	A	ZSWIM4
280578	rs4959719	6	3E+06	26	157	0.20	3.72e-05	0.14	T	C	B	MYLK4
428554	rs12552851	9	2E+07	26	159	0.21	3.85e-05	0.18	T	C	B	—
164526	rs77302400	3	1E+08	26	159	0.16	5.53e-05	0.07	A	G	B	OSBPL11
329319	rs9322337	6	2E+08	26	159	0.11	5.61e-05	0.05	A	C	A	ESR1
189827	rs78741583	4	1E+07	26	159	0.17	6.08e-05	0.14	A	C	B	LINC01085
716116	rs10073	17	8E+07	26	159	6.78	6.33e-05	0.39	T	C	B	ENGASE
520052	rs75036351	11	8E+07	25	158	0.05	6.41e-05	0.03	T	C	B	PAK1
696244	rs112528454	17	2E+07	26	159	0.02	6.67e-05	0.02	A	G	A	—
771638	rs6076798	20	5E+06	26	158	0.28	6.82e-05	0.30	A	G	B	—
730301	rs75215008	18	5E+07	26	158	0.08	7.39e-05	0.04	A	G	A	ZBTB7C
512182	rs118006893	11	5E+07	25	158	0.05	7.65e-05	0.03	A	G	B	FOLH1
512223	rs117702557	11	5E+07	26	159	0.04	8.14e-05	0.02	A	G	A	—
512358	rs77222734	11	5E+07	26	159	0.04	8.14e-05	0.02	T	G	A	—
511999	rs78948210	11	5E+07	26	159	0.04	8.22e-05	0.02	T	C	A	OR4B1
512034	rs79616850	11	5E+07	26	159	0.04	8.22e-05	0.02	T	C	A	OR4S1
513022	rs117283931	11	6E+07	26	159	0.03	8.26e-05	0.02	T	C	B	—
510228	rs4755869	11	4E+07	26	159	0.13	8.68e-05	0.06	A	G	B	—
512249	rs117472250	11	5E+07	26	157	0.05	8.78e-05	0.02	A	C	A	LOC440040
722389	rs3748429	18	1E+07	26	159	0.22	8.93e-05	0.16	A	C	B	PIEZO2
722390	rs3748428	18	1E+07	26	159	0.22	8.93e-05	0.16	T	C	A	PIEZO2
410077	rs2068025	8	1E+08	26	159	6.14	9.20e-05	0.39	T	C	A	TRPS1

Supplemental Table S28. Objective Response adjusted for sex and R-IPI

snpID	rsID	chr	pos	n0	n1	OR	p	MAF	Allele.A	Allele.B	effect.allele	gene
749113	rs72995377	19	1E+07	25	157	0.10	2.86e-05	0.07	A	G	A	ZSWIM4

771636	rs9653638	20	5E+06	26	158	0.21	3.32e-05	0.24	A	G	A	—
428554	rs12552851	9	2E+07	26	159	0.21	3.56e-05	0.18	T	C	B	—
280578	rs4959719	6	3E+06	26	157	0.20	3.73e-05	0.14	T	C	B	MYLK4
329319	rs9322337	6	2E+08	26	159	0.10	3.95e-05	0.05	A	C	A	ESR1
164526	rs77302400	3	1E+08	26	159	0.16	5.63e-05	0.07	A	G	B	OSBPL11
696244	rs112528454	17	2E+07	26	159	0.02	6.09e-05	0.02	A	G	A	—
189827	rs78741583	4	1E+07	26	159	0.17	6.12e-05	0.14	A	C	B	LINC01085
716116	rs10073	17	8E+07	26	159	6.78	6.51e-05	0.39	T	C	B	ENGASE
771638	rs6076798	20	5E+06	26	158	0.28	6.87e-05	0.30	A	G	B	—
510228	rs4755869	11	4E+07	26	159	0.12	7.12e-05	0.06	A	G	B	—
520052	rs75036351	11	8E+07	25	158	0.05	7.39e-05	0.03	T	C	B	PAK1
730301	rs75215008	18	5E+07	26	158	0.08	7.48e-05	0.04	A	G	A	ZBTB7C
512182	rs118006893	11	5E+07	25	158	0.05	7.50e-05	0.03	A	G	B	FOLH1
512223	rs117702557	11	5E+07	26	159	0.04	8.19e-05	0.02	A	G	A	—
512358	rs77222734	11	5E+07	26	159	0.04	8.19e-05	0.02	T	G	A	—
511999	rs78948210	11	5E+07	26	159	0.04	8.30e-05	0.02	T	C	A	OR4B1
512034	rs79616850	11	5E+07	26	159	0.04	8.30e-05	0.02	T	C	A	OR4S1
756094	rs80203774	19	4E+07	26	159	0.08	8.31e-05	0.04	T	C	A	SIPA1L3
513022	rs117283931	11	6E+07	26	159	0.03	8.31e-05	0.02	T	C	B	—
722389	rs3748429	18	1E+07	26	159	0.22	8.72e-05	0.16	A	C	B	PIEZO2
722390	rs3748428	18	1E+07	26	159	0.22	8.72e-05	0.16	T	C	A	PIEZO2
512249	rs117472250	11	5E+07	26	157	0.04	8.83e-05	0.02	A	C	A	LOC440040
410077	rs2068025	8	1E+08	26	159	6.15	9.31e-05	0.39	T	C	A	TRPS1

References

[67] Thermo Fisher Scientific. Axiom Genotyping Solution - Data Analysis Guide. Tech. rep. 2011.
[68] GENEVA Coordinating Center. GWASTools Data Cleaning. Tech. rep. 2020.
[69] Janey Wiggs. Quality Control Report for Genotypic Data. Tech. rep. 2016.

Table S29. Cox regression analysis between clinical/pathological characteristics and PFS or OS (n=185)

	PFS			OS		
**Predictors**	**HR**	**CI**	**p**	**HR**	**CI**	**p**
**Sex : Female**	0.34	0.17 – 0.69	0.003	0.40	0.17 – 0.91	0.029
**Age**	1.03	1.00 – 1.05	0.067	1.04	1.00 – 1.08	0.037
**Stage: 3-4**	3.12	1.48 – 6.59	0.003	3.82	1.44 – 10.14	0.007
**R-IPI: Very good (0)**	0.46	0.11 – 1.99	0.299	0.32	0.04 – 2.47	0.276
**R-IPI: Poor (3-5)**	2.55	1.34 – 4.88	0.005	2.22	1.01 – 4.87	0.047

Table S30. Logistic regression analysis between clinical/pathological characteristics and objective response (n=185)

	Objective response		
**Predictors**	**OR**	**CI**	**p**
**Sex : Female**	1.18	0.51 – 2.75	0.694
**Age**	0.97	0.93 – 1.00	0.069
**Stage: 3-4**	0.39	0.15 – 0.95	0.046
**R-IPI: Very good (0)**	0.58	0.18 – 2.26	0.391
**R-IPI: Poor (3-5)**	0.37	0.14 – 0.92	0.032

Table S31. SNPs associated with PFS or OS at the highest p-values

SNPs	*TNIP3* rs116665727	*LNC00882* rs1607795	*CIDEA* rs75614943	*NOS1* rs77241831	*CSRP3-AS1* rs78466241	*SLC35F4* rs74832512	*PPP6R3* rs117500207	*CDH3* rs35789195	*OXNAD1* rs11721010	*LYZL1* rs12356569
**PFS**										
Primary analysis	$p=1.0*10^{-9}$	$p=1.3*10^{-8}$	$p=1.9*10^{-8}$	$p=3.1*10^{-8}$						
R-IPI adjusted			$p=7.0*10^{-9}$	$p=3.1*10^{-8}$			$p=4.5*10^{-8}$			
Sex adjusted										
R-IPI and sex adjusted					$6.9*10^{-9}$					[$p=5*10^{-8}$]
**OS**										
Primary analysis						$p=1.7*10^{-8}$				
R-IPI adjusted						$p=7.0*10^{-9}$	$p=2.6*10^{-8}$	$p=4.9x10^{-8}$		
Sex adjusted						$p=2.3*10^{-8}$			$p=2.4*10^{-8}$	
R-IPI and sex adjusted						$p=3.4*10^{-8}$				$p=4.0*10^{-8}$

[ ] very high trend
